# A universal oxygen scavenger for oxidase-based biosensors

**DOI:** 10.1126/sciadv.adw6133

**Published:** 2025-09-12

**Authors:** Huijie Zhang, Mohamed G. Saadeldin, Darren Buesen, Hamzah Elfaitory, Jakob Burger, Vincent M. Friebe, Jonas Honacker, Tobias Vöpel, Alaa A. Oughli, Nicolas Plumeré

**Affiliations:** ^1^ School of New Energy, Nanjing University of Science and Technology, Jiangyin, Jiangsu 214443, China.; ^2^ Professorship for Electrobiotechnology, Campus Straubing for Biotechnology and Sustainability, Technical University of Munich, Uferstraße 53, 94315 Straubing, Germany.; ^3^ Laboratory of Chemical Process Engineering, Campus Straubing for Biotechnology and Sustainability, Technical University of Munich, Uferstraße 53, 94315 Straubing, Germany.

## Abstract

Oxidase-based electrochemical biosensors are widely deployed for point-of-use applications, yet oxygen interference remains a major challenge, substantially reducing sensing accuracy. Here, we developed a universal enzymatic O_2_ scavenger composed of alcohol oxidase, catalase, and paraformaldehyde to eliminate O_2_ within the sensor by converting it to water. Unlike other oxidases, alcohol oxidase exclusively uses O_2_ as an electron acceptor, preventing interference with the electron transfer chain involving the sensing oxidase. We demonstrated the compatibility of this O_2_ scavenger for calibration-free sensing of glucose, lactate, and creatinine in the concentration range relevant to human health. Without the O_2_ scavenger, sensor readings were less than 50% of those under inert gas conditions. With the O_2_ scavenger, the accuracy improved to 99%, even at low substrate concentrations. The general compatibility and performances of this alcohol oxidase-based O_2_ scavenger unlock the full potential of oxidase-based biosensors for reliable point-of-use sensing.

## Teaser

Alcohol oxidase’s unique selectivity for O_2_ enables effective elimination of O_2_ interference, unlocking accurate biosensing.

## INTRODUCTION

Electrochemical biosensors based on redox enzymes are extensively used for providing quantitative information in food analysis and health care (*1*, *2*). Their simplicity, extreme selectivity, and potential for analyte diversification (*3*) make them highly attractive for point-of-use sensing, as exemplified by the glucose biosensor (*4*–*7*), produced on a scale exceeding 10^10^ units annually (*8*, *9*). Redox enzymes, used as biorecognition elements, catalyze the oxidation or reduction of specific analytes, generating an electrical current that serves as the sensing signal. Despite the high specificity of these enzymes, interferences in the electron transfer chain often reduce the accuracy of the analyte quantification and require additional calibration steps.

**Fig. 1.**
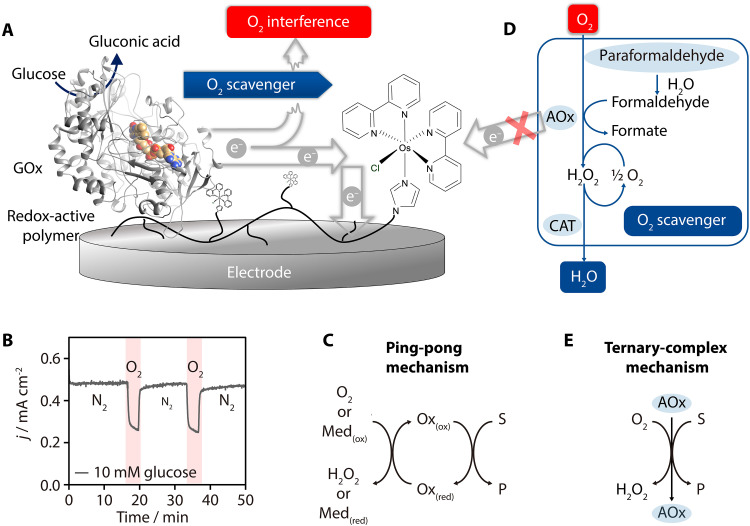
Oxidase-based biosensing and oxidase-based O_2_ scavenging. (**A**) Amperometric glucose biosensor based on GOx as a biorecognition element and an Os complex–modified polymer for the immobilization and wiring of GOx to the electrode. (**B**) When subjected to O_2_, GOx reduces O_2_, diverting electron flow and thus decreasing the anodic current from glucose oxidation. (**C**) Ping-pong mechanism of classical oxidases (Ox). S is the substrate, and P is the product. (**D**) The O_2_ scavenger based on AOx oxidizes formaldehyde generated from the depolymerization of paraformaldehyde to formate and thus reduces O_2_ to H_2_O_2_, which is further reduced to water through the combined reactions of catalase and AOx. AOx may use either formaldehyde as the substrate to produce formate or short-chain, primary, aliphatic alcohols to produce aldehydes. (**E**) Ternary complex mechanism of AOx, showcasing simultaneous docking of both oxygen and the alcohol substrate to realize highly specific oxygen scavenging.

The most ubiquitous interference is molecular oxygen, which is difficult to avoid in point-of-use sensing due to measurements being conducted in ambient air or oxygen-rich media such as blood (*10*). Oxygen can interfere with all types of oxidoreductase-based electrochemical biosensors through various mechanisms. For reductase-based sensors (*11*–*16*), enzymatic analyte reduction typically requires electrode potentials that are more negative than the potential for the O_2_ reduction reaction. As a result, O_2_ is reduced directly at the electrode, leading to an overestimation of the analyte concentration (*12*–*16*). In contrast, in oxidase-based sensors, the oxidase intrinsically uses O_2_ as a natural electron acceptor (*2*). This property of oxidases was exploited in first-generation electrochemical biosensors, which were poised to detect the product of O_2_ reduction by the oxidase hydrogen peroxide (*2*). However, this O_2_ dependency inherently limits the sensor’s performance, especially at high substrate concentrations (*17*). In second-generation biosensors, artificial electron mediators are used to accept the electrons from the oxidase and shuttle them to the electrode, theoretically making the sensing independent of O_2_ (*2*, *18*). Nevertheless, O_2_ still competes with the artificial mediators for the electrons generated by the oxidase (*19*, *20*), diverting them away from the electrode (Fig. 1A). The result is a decrease in the current signal, which leads to an underestimation of the analyte concentration (Fig. 1B) (*21*).

Many approaches have been proposed for sample deoxygenation to bypass the adverse effect of O_2_ (*12*). Vacuum degassing and inert gas purging (*22*, *23*) are practical in a lab environment but incompatible with point-of-use applications. Alternatively, reducing agents, such as ascorbic acid (*24*), sodium thiosulfate (*25*), and sodium sulfite in the presence of a catalyst (*26*), can be used for the removal of O_2_. However, their intrinsic electroactivity, or the electroactivity of the required catalyst, often introduces additional interferences (*26*, *27*). Moreover, such chemical methods are often insufficiently fast for oxidase-based biosensors where the presence of O_2_ leads to a competing electron transfer process even before an electrical potential is applied.

Fast O_2_ scavenging can be achieved with the use of enzymatic O_2_ scavengers. The most commonly used is glucose oxidase (GOx) coupled with catalase (CAT) in the presence of glucose as a reducing agent, leading to effective O_2_ scavenging for many applications (*28*–*30*). It is generally compatible with electrochemical biosensors based on reductases (*11*, *31*) but typically incompatible with oxidase-based biosensors. The first issue is an overlap in substrate specificity between the oxidase used for O_2_ scavenging and the oxidase used for sensing. This problem can often be mitigated, given the vast biodiversity that offers a wide range of highly specific oxidases. The second, more challenging issue remains unresolved: While oxidases are generally specific to their substrate, they tend to be highly promiscuous with electron acceptors. The catalytic mechanism of oxidases typically follows a so-called ping-pong mechanism (Fig. 1C) (*32*, *33*), where the enzyme is first reduced by the substrate and then reoxidized, either by its natural electron acceptor (O_2_) or by artificial electron mediators designed to shuttle electrons in second-generation electrochemical biosensors (*2*). Consequently, artificial electron acceptors, such as Os complexes (*9*) or ferrocene derivatives (*5*, *34*) commonly used in oxidase-based biosensors, may also accept electrons from the oxidase used for O_2_ scavenging. This can interfere with the anodic electron transfer involved in the sensing function.

Here, we develop a universal enzymatic O_2_ scavenger that is compatible with oxidase-based electrochemical biosensors using alcohol oxidase (AOx) and catalase. This system catalyzes the reaction between a reducing agent (paraformaldehyde or ethanol) and O_2_ to yield water as the sole product from O_2_ reduction (Fig. 1D). We selected AOx as a catalyst for O_2_ scavenging because it exclusively uses O_2_ as an electron acceptor owing to a ternary-complex mechanism requiring simultaneous O_2_ and substrate binding in the catalytic active site for substrate oxidation (Fig. 1E) (*33*). This unique characteristic among oxidases ensures that no other natural or synthetic electron acceptors can capture electrons from AOx, conferring crucial specificity in O_2_ scavenging systems that are currently lacking in biosensors using oxidases. A second useful feature of AOx is that in addition to catalyzing the oxidation of short-chain primary aliphatic alcohols to aldehydes and H_2_O_2_ using O_2_, it also catalyzes the oxidation of formaldehyde to formate (*35*). Given that formaldehyde is rapidly generated in situ from the depolymerization of paraformaldehyde (*36*), we used the latter as a nonvolatile precursor for the substrate of AOx. This greatly facilitates the handling of the sensors for point-of-care applications.

## RESULTS

### AOx as a catalyst for O_2_ scavenging

We screened several AOxs from different organisms and selected the one from *Pichia pastoris* because of its superior activity (table S1). This enzyme exhibited the highest catalytic turnover (*k*_cat_) with methanol (161.7 ± 7.9 s^−1^) as the substrate. The *k*_cat_ decreased with increasing alkyl chain length of the alcohol (e.g., *k*_cat_ = 121.3 ± 11.8 s^−1^ for ethanol). However, because of their volatility, short-chain alcohols are not practical as reducing agents for the long-term storage of the sensors. AOx also catalyzes the reaction of formaldehyde with O_2_ to produce formate (*k*_cat_ = 13.5 ± 0.1 s^−1^; table S1) (*35*). While formaldehyde is also highly volatile, its polymeric form, paraformaldehyde, is a solid that rapidly depolymerizes to formaldehyde upon addition of an aqueous solution. Hence, it can serve as a nonvolatile precursor for formaldehyde for the AOx-based O_2_ scavenger.

**Fig. 2.**
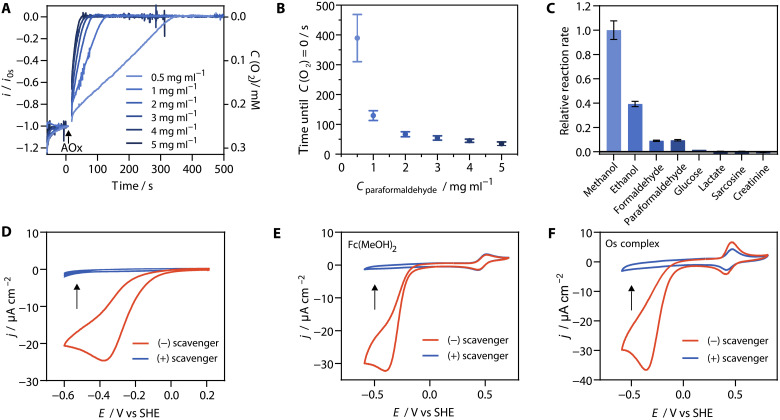
O_2_ removal using AOx, catalase, and paraformaldehyde. (**A**) Monitoring of the current response and O_2_ concentration over time with a carbon microelectrode (calibrated for O_2_ sensing) for different concentrations of paraformaldehyde used as a reducing agent for O_2_ scavenging. CAT was initially present in the solution. AOx was added at 0 s, and the solution was stirred for 10 s. The current responses are normalized to their initial value at 0 s. (**B**) Time needed until the O_2_ reduction current vanishes for different concentrations of paraformaldehyde. (**C**) Relative reaction rates of AOx-catalyzed oxidation with different reducing agents (used at 10 mM) determined from an enzymatic assay. (**D**) CVs (2 mV s^−1^) at a GCE in an unstirred electrolyte (1 ml) with AOx and CAT (red trace) and 20 successive CVs upon addition of paraformaldehyde (5 mg ml^−1^, blue trace). (**E**) CVs (2 mV s^−1^) of a GCE in an electrolyte (1 ml) with AOx, CAT, and Fc(MeOH)_2_ (100 μM, red trace) and upon addition of paraformaldehyde (5 mg ml^−1^, blue trace). (**F**) CVs (10 mV s^−1^) of a GCE modified with the Os complex–modified polymer with AOx and CAT (red trace) and upon addition of paraformaldehyde (5 mg ml^−1^, blue trace). All experiments were performed in phosphate buffer (100 mM, pH 7.5), and the scavenger contains AOx (10 U ml^−1^) and CAT (2000 U ml^−1^).

The effect of the paraformaldehyde concentration on the reaction rate of O_2_ removal was evaluated using a carbon microelectrode to monitor the O_2_ concentration. The O_2_ level of the aerated solutions before AOx addition was ~0.25 mM. Upon addition of AOx into solutions containing paraformaldehyde and catalase, the O_2_ reduction current rapidly decreased until complete O_2_ depletion, whereby depletion rates increased as the paraformaldehyde concentration increased (Fig. 2A). The time required for the O_2_ reduction current to disappear decreased from 389 to 67 s when increasing the concentration of paraformaldehyde from 0.5 to 2 mg ml^−1^ (Fig. 2B). At higher paraformaldehyde concentrations, the time for O_2_ depletion decreased only moderately (35 s for complete O_2_ removal at a paraformaldehyde concentration of 5 mg ml^−1^), indicating that saturation of paraformaldehyde was reached (*37*). At saturating paraformaldehyde concentrations, the rate of O_2_ reduction catalyzed by AOx is similar to that obtained with 10 mM formaldehyde (Fig. 2C). This rapid O_2_ depletion indicates that the rate of depolymerization of paraformaldehyde and the subsequent oxidation of formaldehyde are fast, making paraformaldehyde a suitable reducing agent for the AOx-based O_2_ scavenger.

The AOx-based O_2_ scavenger system was further tested for its ability to maintain anaerobic conditions. The presence of O_2_ was qualitatively monitored by cyclic voltammetry using a glassy carbon electrode (GCE) as the working electrode in a small-volume (1 ml) electrochemical cell open to ambient air. Before the addition of the O_2_ scavenging system, O_2_ reduction appears at a potential lower than −0.1 V versus the standard hydrogen electrode (SHE) on the GCE as a large irreversible cathodic current response (Fig. 2D and fig. S1). After the addition of the O_2_ scavenging system into the solution, the O_2_ reduction signal disappears, which confirms the effective O_2_ removal (Fig. 2D and fig. S1A). With paraformaldehyde (5 mg ml^−1^), the anaerobic condition was maintained for at least 4 hours in solutions exposed to air, as demonstrated by the absence of any O_2_ reduction signal in the 20 successive cyclic voltammograms (CVs) (Fig. 2D). Similar results were obtained using EtOH as the reducing agent instead of paraformaldehyde (fig. S1B).

### Potential sources of interference and inactivation

In addition to establishing and sustaining anaerobic conditions, the application of the AOx-based O_2_ removal system in biosensing requires that neither its components nor the byproducts of the O_2_ reduction reaction interfere with the electron transfer chain driving the catalytic reaction for analyte detection. Such interferences may originate from (i) the reaction between the analyte of the biosensor (e.g., glucose, lactate, or creatinine) and AOx, (ii) the reaction between AOx and the electron mediators, or (iii) the reaction involving the oxidation products of the reducing agent (e.g. aldehydes and formate).

We used a glucose biosensor as a case study for the integration of the AOx-based scavenger. Commercial glucose biosensors typically rely on either GOx or glucose dehydrogenases. While glucose dehydrogenases are O_2_-insensitive, their broader substrate specificity can lead to measurement errors (*38*). In contrast, GOx exhibits high specificity for glucose but is susceptible to interference from O_2_ (*18*, *39*, *40*). In commercial GOx-based biosensors, O_2_ interference is typically addressed through factory calibration by assuming a fixed physiological O_2_ level. However, O_2_ level fluctuations due to hypoxia, perfusion, or high-altitude condition (*41*) can introduce measurement errors even after calibration (*42*). This makes the GOx-based biosensors an ideal target for the implementation of the AOx-based O_2_ scavenger.

Glucose has a primary alcohol group in its open-chain form, making it a potential substrate for AOx. In comparison to other primary aliphatic alcohols and to formaldehyde at the same concentration, the reaction rate of glucose with AOx in the presence of O_2_ is negligible (Fig. 2C). Other classical substrates targeted in oxidase-based biosensing, such as lactate or creatinine as well as sarcosine (an intermediate produced during creatinine sensing), likewise do not lead to any significant oxidation by AOx in the presence of O_2_. Hence, the high substrate specificity of AOx for short-chain primary alcohols and formaldehyde as reducing agents (*35*) ensures that analytes such as glucose, lactate, or creatinine are not affected by the AOx-catalyzed O_2_ removal system.

The possibility of interfering reactions between AOx and mediators was tested with the freely diffusing ferrocenedimethanol [Fc(MeOH)_2_] and with a polymer-bound Os complex [(poly(1-vinylimidazole)-Os(bpy)_2_Cl (*43*)] (Materials and Methods and fig. S2) that are classically used for electron mediation in oxidase-based biosensing (Fig. 2, E and F). Before the addition of paraformaldehyde in the electrolyte containing AOx, catalase, and Fc(MeOH)_2_, the CV displays the irreversible current wave for O_2_ reduction as well as the reversible currents as a result of Fc(MeOH)_2_ oxidation and reduction. After the addition of paraformaldehyde, which completes the O_2_ scavenging system, the O_2_ reduction wave disappears, while the current response for Fc(MeOH)_2_ remains unchanged (Fig. 2E). In the case of the Os complex–modified polymers, before the addition of paraformaldehyde into the electrolyte containing AOx and catalase, the CV also shows the O_2_ reduction peak and the redox peaks of the Os complex (Fig. 2F). After the addition of paraformaldehyde, the O_2_ reduction peak disappears and the CV still only shows the reversible redox peaks of the Os complex. The absence of catalytic current in these experiments with either Fc(MeOH)_2_ or the Os complex–modified polymers demonstrates the exclusive substrate specificity of AOx for O_2_ as the electron acceptor. This ensures that artificial electron mediators used in oxidase-based biosensors are not affected by the AOx-catalyzed O_2_ removal. In addition, the absence of any oxidation current in Fig. 2 (E and F) shows that paraformaldehyde is not electroactive within the sensing potential window. In contrast, reducing agents such as ascorbic acid can undergo direct oxidation at the electrode surface, leading to interference (*27*). Similarly, sodium thiosulfate and sodium sulfite require catalysts (e.g., transition metals) for efficient O_2_ removal, which may introduce further interference (*26*, *44*).

The products of the AOx-based O_2_ scavenger are aldehydes resulting from the oxidation of the primary alcohols or formic acid resulting from the oxidation of formaldehyde when paraformaldehyde (or methanol) is used as the substrate. The influence of the products on the activity of GOx was monitored by recording the catalytic current for glucose oxidation over time in an electrolyte containing glucose, GOx, Fc(MeOH)_2_, and the AOx-based O_2_ scavenging system. After 3 hours, the catalytic current decreased only slightly when using an alcohol as the substrate for AOx (fig. S3A). These current decreases are comparable to the ones obtained in the absence of the O_2_ scavenging system when purging the electrolyte with Ar to remove O_2_. When using paraformaldehyde as the substrate, the catalytic current for glucose oxidation steadily decreases over time, which closely follows the pH drop from 7.1 to 6.7 because of the generation of formic acid (fig. S3B). Nevertheless, this pH and activity decay is on the timescale of hours. Oxidase-based sensors for point-of-care applications are single-use devices operated at the timescale of seconds (current readout) or minutes (charge readout). Hence, these moderate activity changes induced by the products of the AOx-based O_2_ scavenger are not critical with respect to the glucose biosensing applications. These experiments also demonstrate that near-saturating concentrations of paraformaldehyde do not induce major deactivation of GOx.

### AOx-based O_2_ removal for glucose biosensing

The performance of the O_2_ scavenger was tested on a glucose biosensor based on GOx. The experiments were first conducted in a standard three-electrode cell with a GCE as a working electrode to demonstrate the proof of concept for glucose sensing. In a first sensing configuration, the redox mediator Fc(MeOH)_2_ and GOx were freely diffusing in solution. In a second configuration, GOx was immobilized on the electrode in the Os complex–modified polymer (*43*, *45*). In the latter case, the electrode was modified with a mixture containing polymer (50 wt %), GOx (40 wt %), and a cross-linker (10 wt %) at a total loading of 200 μg cm^−2^. This ratio and loading led to the maximum catalytic currents for glucose oxidation (fig. S4).

**Fig. 3.**
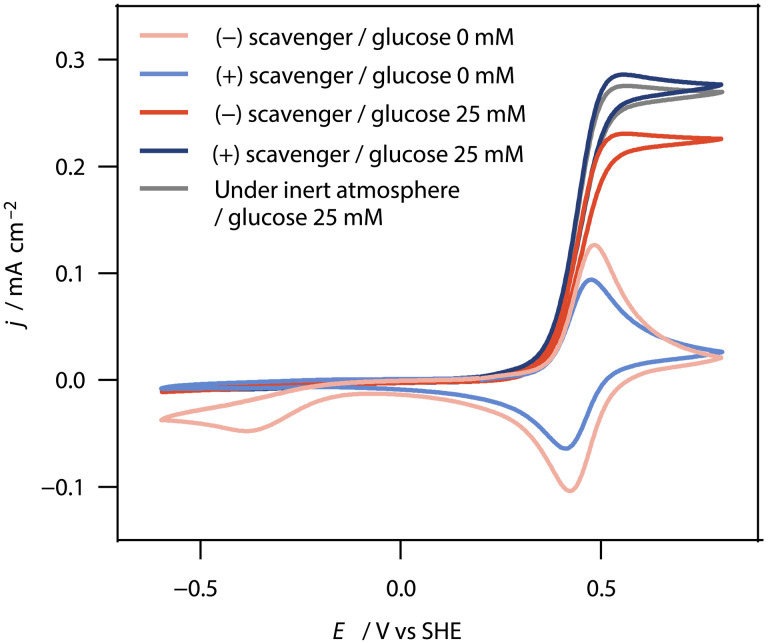
Glucose sensing with modified GCEs. CVs (10 mV s^−1^) of modified GCEs. Nonturnover CVs (0 mM glucose) in the presence of AOx and CAT only (pink trace) and upon addition of paraformaldehyde (light blue trace). CVs with glucose (25 mM) in the presence of AOx and CAT (red trace), upon addition of paraformaldehyde (dark blue trace), and in the absence of any component of the scavenger but under an inert atmosphere (gray trace). All experiments were performed in phosphate buffer (5 ml, pH 7.5, 100 mM) exposed to ambient air, unless stated otherwise. The scavenger contains AOx (10 U ml^−1^), CAT (2000 U ml^−1^), and paraformaldehyde (5 mg ml^−1^). The GCEs were modified with the Os complex–modified polymer (50 wt %), GOx (40 wt %), and PEGDGE (10 wt%) with a total loading of 200 μg cm^−2^.

To confirm the absence of unwanted cross-reactions between GOx and the substrates of AOx, we conducted experiments involving GOx, AOx, CAT, the reducing agent for AOx (paraformaldehyde or ethanol), and either the electron mediators Fc(MeOH)_2_ (fig. S5) or the Os complex–modified polymer (Fig. 3). The resulting CVs show solely the reversible redox wave of the mediator. The absence of a catalytic current demonstrates that GOx does not oxidize ethanol or paraformaldehyde.

A catalytic current was observed only after the addition of glucose (25 mM) to the buffer, demonstrating catalytic glucose oxidation by GOx and electron transfer from GOx to the electrode via the Os complexes (Fig. 3) or Fc(MeOH)_2_ (fig. S5, A and B). Notably, the catalytic current in the presence of the O_2_ scavenger was higher compared to currents obtained under ambient conditions without the scavenger and closely matched the current observed under an inert atmosphere. This demonstrates that the O_2_ scavenger effectively suppresses O_2_ interference in glucose sensing.

### AOx-based O_2_ removal for glucose biosensing in a single-use screen-printed electrode

**Fig. 4.**
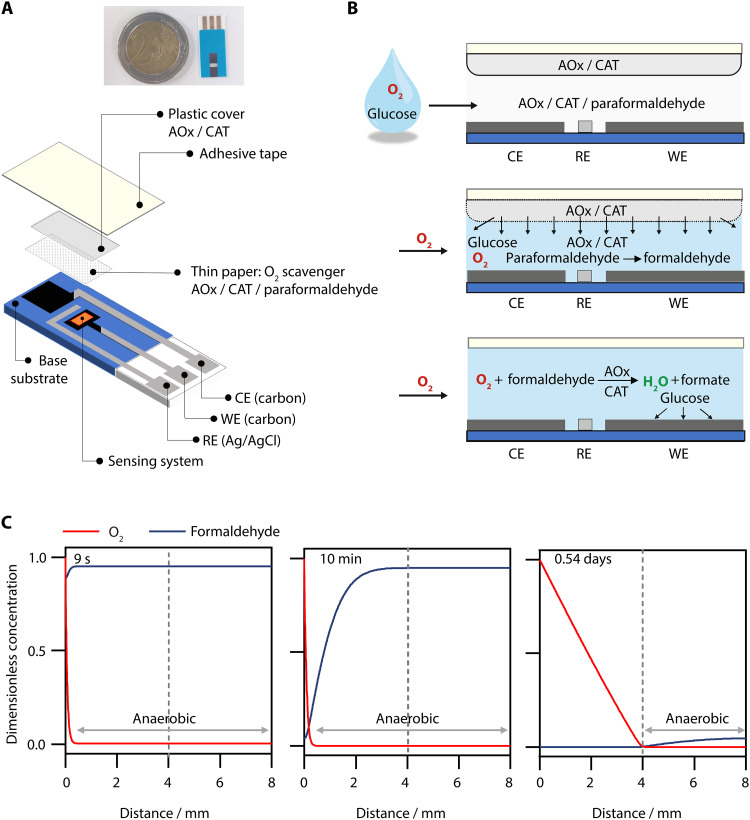
SPE assembly and simulations of O_2_ penetration into the sensor. (**A**) Assembly of a single-use glucose biosensor on an SPE with the oxygen scavenger in the self-filling capillary channel (~3 μl). The working electrode (WE) and counter electrodes (CEs) are made of carbon, and the pseudo-RE is made of Ag/AgCl paste. (**B**) The filling of the capillary channel with the aqueous sample leads to the dissolution of the components of the O_2_ scavenger followed by the depletion of O_2_. (**C**) Simulations of the O_2_ concentration and formaldehyde concentration within the capillary channel when using paraformaldehyde as the reducing agent after 9 s (time needed for depletion of the O_2_ initially dissolved in the sample), after 10 min (time used for the coulometric measurements), and after 0.54 days (time needed for O_2_ to reach the sensing electrode). The gray dashed line shows the position of the sensing electrode. The simulations account for the continuous supply of O_2_ through the capillary opening exposed to ambient air. The parameter values for the simulation are given in table S2.

Glucose biosensor strips for point-of-care applications typically consist of screen-printed electrodes (SPEs) with a small volume capillary channel for sample loading. To integrate the enzymatic O_2_ scavenger into these sensor strips, the electrode layout and capillary channel were specifically designed to create and maintain anaerobic conditions at the sensing electrode (Fig. 4A). The pseudo–reference electrode (pseudo-RE) is made of Ag/AgCl, which has a potential of 0.13 V versus the SHE determined by using the redox signal of the Os complex–modified polymer as a reference (fig. S6). The potential of the pseudo-RE remained stable over a 24-hour period, as demonstrated by the consistent peak potentials of the Os complex–modified polymer observed in successive CVs, with no influence from the presence of O_2_ (fig. S7, A and B). CAT and AOx were deposited on the inner surface of the capillary cover, while paraformaldehyde was distributed in solid form on a thin paper serving as a spacer within the channel (fig. S8). In comparison to the experiments in the large electrochemical cell (Fig. 3), the concentrations of the scavenger components were increased 4-fold (AOx) to 10-fold (paraformaldehyde) to account for concentration heterogeneities within the capillary upon filling with the sample. The unit ratio between AOx and CAT was 1:100. The large excess in CAT activity is used to ensure that the H_2_O_2_ generated by AOx is rapidly consumed, given that it may deactivate the sensing enzymes (*2*, *46*–*48*). This is the typical ratio used for O_2_ scavenging (*11*). The addition of a 10-fold excess of paraformaldehyde did not lead to an accelerated decline in catalytic current for glucose oxidation catalyzed by GOx (fig. S9), making it compatible with glucose sensing. GOx and the Os complex–modified polymer were drop-cast onto the working (sensing) electrode, leading to a film with reproducible homogeneity in thickness (the standard deviation corresponding to the film thickness distribution is 55% ± 12%; *n* = 3; fig. S10). When the aqueous sample fills the channel, the components of the O_2_ scavenger dissolve and spread throughout the channel, initiating O_2_ depletion (Fig. 4B).

A key aspect of this design is that the sensing electrode is placed at the end of a channel long enough (>4 mm) to ensure that O_2_ is fully reduced by the O_2_ scavenger system before it can diffuse from the capillary openings to the sensing electrode. Simulations of the O_2_ scavenging process (Supplementary Materials, figs. S11 to S16) predict that O_2_ within the sample is completely depleted 9 s after introduction into the capillary channel when using paraformaldehyde and 1.1 s when using ethanol (Fig. 4C and fig. S17). Simulations of the O_2_ and paraformaldehyde concentration profiles within the capillary for a typical sensing duration in the coulometric mode (about 10 min) predict that O_2_ penetrates less than 0.5 mm into the capillary, ensuring that glucose sensing occurs under local anaerobic conditions at the sensing electrode. The simulations also predict that it would take 0.54 days (or 0.95 days when using 50 mM EtOH as the reducing agent) for O_2_ entering through the capillary opening to deplete the reducing agent and reach the sensing electrodes. To validate these calculations experimentally, we monitored the O_2_ reduction current over time by cyclic voltammetry at SPEs containing the O_2_ scavenger (fig. S7). The O_2_ reduction signal appears ~4 hours after the addition of buffer and constant exposure to air. While about three times shorter than the theoretically predicted value of 13 hours, the O_2_-free duration remains in the order of hours. The difference between the experimentally observed and theoretically predicted O_2_-free durations is likely due to the gradual dissolution and depolymerization of paraformaldehyde, leading to spatial and temporal heterogeneity in the formaldehyde concentrations within the capillary channel. These timescales of hours far exceed the typical sensing duration of single-use electrochemical biosensors (minute timescale), making this sensor and scavenger design compatible with virtually any point-of-use sensing application (Fig. 4C and fig. S17).

**Fig. 5.**
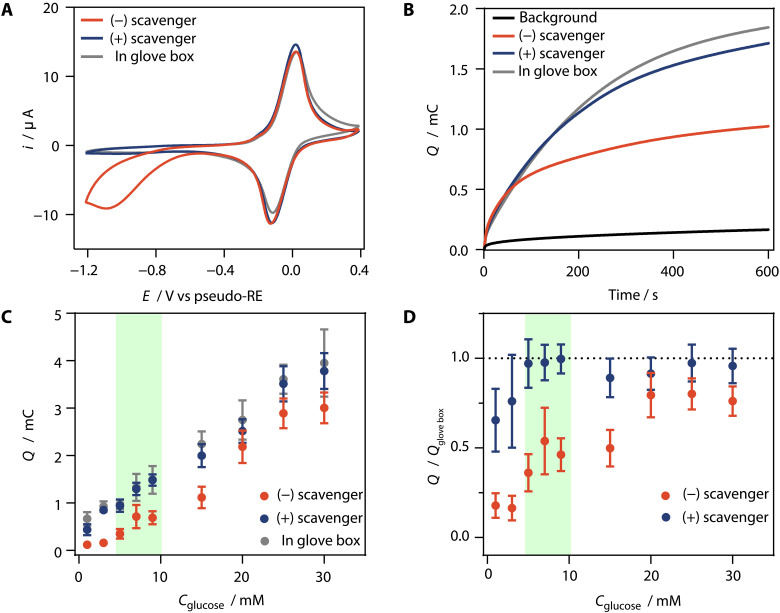
Glucose sensing with the SPE. (**A**) CVs (10 mV s^−1^) of SPEs modified with GOx, redox-active polymer, and paraformaldehyde in the absence of AOx and CAT (red trace), in the presence of all components of the scavenger (blue trace), and in the presence of all components of the scavenger under an inert atmosphere (gray trace). (**B**) Chronocoulometry of the modified SPE with paraformaldehyde but in the absence of AOx and CAT without glucose (black trace) and with 9 mM glucose (red trace), in the presence of all components of the scavenger with 9 mM glucose (blue trace), and with all components of the scavenger under an inert atmosphere with 9 mM glucose (gray trace). (**C**) Charge versus glucose concentrations in the presence of paraformaldehyde only (red circles), in the presence of all components of the scavenger (blue circles), and with all components of the scavenger under an inert atmosphere (gray circles). (**D**) Normalized charge versus glucose concentration. The charge measured under inert conditions was used for normalization. The green area indicates the normal glucose range for humans. All samples consisted of phosphate buffer (pH 7.5, 100 mM) with glucose concentrations as indicated. All experiments were performed in ambient air, unless stated otherwise. The SPEs were modified with the Os complex–modified polymer (50 wt %), GOx (40 wt %), and PEGDGE (10 wt %) with a total loading 200 μg cm^−2^. The scavenger containing AOx (40 U ml^−1^), CAT (4000 U ml^−1^), and paraformaldehyde (saturating concentrations ~50 mg ml^−1^) is integrated within the capillary channel of the SPE. The applied potential is 0.4 V versus the pseudo-RE in the SPE. Data are the average of *n* = 3 repetitions, and error bars show the standard deviation.

The effect of the AOx-based scavenger on O_2_ removal within this capillary channel of the sensor strip was evaluated by cyclic voltammetry. In the absence of the O_2_ scavenger, the O_2_ reduction signal appears at a potential lower than −0.45 V versus the pseudo-RE (Fig. 5A). In contrast, when the AOx-based O_2_ scavenger was added, the signal of O_2_ reduction is fully suppressed in the CV, which is identical to the CV performed inside a glove box. No waiting time was used between the sample addition and the start of the CV. This demonstrates that the anaerobic conditions are achieved quickly within the timescale of the experiment (about 100 s until the potential reaches the O_2_ reduction potential), which is in excellent agreement with the prediction from the simulations.

The effect of the O_2_ scavenger on glucose sensing in the self-filling sensor strips was assessed by means of chronocoulometry (Fig. 5B) over a wide glucose concentration range pertinent to blood glucose levels (Fig. 5C). The charge obtained under an inert atmosphere was used for normalization of the charge obtained under air and in the presence of the O_2_ scavenger (Fig. 5D). The charges measured in ambient air without the O_2_ scavenger were lower than those measured under an inert atmosphere, particularly at glucose concentrations below 20 mM. The effect was less pronounced at higher concentrations because of the limited availability of O_2_ in the small volume capillary and the hindered replenishment of O_2_ from ambient air through the capillary channel. In the presence of the O_2_ scavenger, the charge values show excellent agreement with those obtained under an inert atmosphere for glucose concentrations above 5 mM. At the glucose concentration in the low millimolar range, the measured charges were still lower than those observed under anaerobic conditions, although higher than those measured in the absence of the scavenger. This is because the O_2_ scavenger components require some time to dissolve and fully remove O_2_ within the capillary channel of the sensor. During this initial phase, residual O_2_ can react with reduced GOx, leading to a fraction of the electrons generated from glucose oxidation being diverted to O_2_. Nevertheless, the glucose readings in the presence of the scavenger are highly accurate in the normal concentration range for humans (4 to 10 mM, green area in Fig. 5, C and D) (*49*) and in the high concentration range, which is the most relevant to diabetes management with respect to the need for insulin intake (*50*). The biosensor with the O_2_ scavenger exhibits a sensitivity of 114.5 μC mM^−1^, with a detection limit of 0.72 mM (fig. S18).

The glucose sensing strip equipped with the O_2_ scavenger was tested in both amperometric and coulometric modes under various temperatures (fig. S19). For the same glucose concentrations (12 mM), the amperometric mode delivers a standard deviation of 19.6% on average, while the coulometric mode has an average standard deviation of 7.5% in the temperature range from 5° to 45°C (table S4). The amperometric operation has a fast response time but is more dependent on external parameters such as temperature, which influence the enzyme activity or mass transport and thus lead to fluctuations in the current reading. In contrast, the electrical charge extracted in the coulometric mode is highly reproducible, regardless of the external temperature. This demonstrates the possibility for calibration-free sensing with oxidase-based sensors in ambient air.

### Blood analysis

**Fig. 6.**
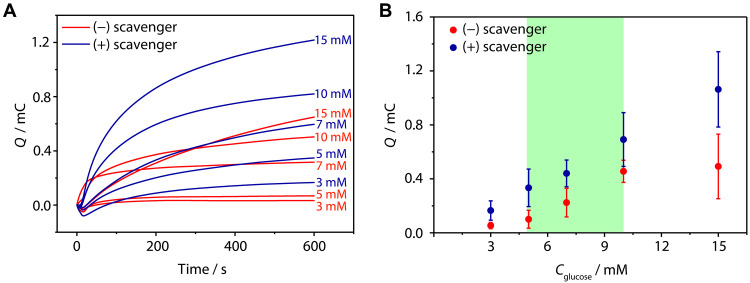
Blood sample analysis using glucose spiking. (**A**) Chronocoulometric analysis of modified SPEs in ambient air with paraformaldehyde only (red) and with all components of the O_2_ scavenger (blue). (**B**) Charge versus glucose concentration of modified SPEs in ambient air with paraformaldehyde only (red) and with all components of the O_2_ scavenger (blue). *E*_app_ = 0.6 V versus the pseudo-RE for 10 min. Glucose (3, 5, 7, 10, and 15 mM) was added to the blood. Data collected without glucose addition were used for baseline correction. The SPEs were modified with the Os complex–modified polymer (50 wt %), GOx (40 wt %), and PEGDGE (10 wt %) with a total loading of 200 μg cm^−2^. The scavenger containing AOx (40 U ml^−1^), CAT (4000 U ml^−1^), and paraformaldehyde (saturating concentrations ~50 mg ml^−1^) is integrated within the capillary channel of the SPE. For (B), data are the average of *n* = 3 repetitions, and error bars show the standard deviation.

For validation under point-of-use conditions, we tested the glucose sensing in a human blood sample with the GOx-based sensors and the integrated AOx-based O_2_ scavenger (Fig. 6). The blood was tested without any treatment. Because of the presence of chloride ions in the sample matrix (blood), which shifts the potential of the pseudo-RE of the SPEs (fig. S6), we applied a higher potential (0.6 V versus the pseudo-RE) compared to the measurements conducted in buffer (*51*). The charges obtained for the blood sample (1145 ± 201 μC without the scavenger and 1212 ± 165 μC with the scavenger) were similar to those obtained when measuring buffer only (1050 ± 109 μC without the scavenger and 988 ± 121 μC with the scavenger), indicating that the glucose concentration is near zero. To test the sensing of higher glucose concentration, we spiked the blood sample with specific glucose concentrations (3 to 15 mM). Without the O_2_ scavenger, the obtained charges are low and nonlinear with respect to glucose concentration (Fig. 6). In contrast, in the presence of the integrated O_2_ scavenger, the electrical charges are higher and proportional to the glucose concentrations. This demonstrates that the O_2_ scavenger is effective for suppressing O_2_ interference even in complex sample matrices such as blood, qualifying it for point-of-care sensing applications.

### Beyond the glucose biosensor

The properties of the AOx-based O_2_ scavenger make it theoretically compatible with all oxidase-based biosensors, except those targeting short-chain primary alcohols, formaldehyde or formate, which are substrates and products of the AOx-catalyzed reactions. To verify this versatility, we tested two additional oxidase-based biosensors for lactate and creatinine, which are among the most relevant clinical analytes after glucose (*52*, *53*), both of which are typically prone to O_2_ interferences (*54*). Lactate oxidase can be wired to an electrode via redox-active polymers (*34*). Here, we constructed a lactate biosensor by immobilizing lactate oxidase on a GCE using the same Os complex–modified polymer used for the glucose biosensor. Likewise, we developed a creatinine biosensor by immobilizing sarcosine oxidase in the same redox-active polymer and combining it with creatininase and creatinase in solution.

**Fig. 7.**
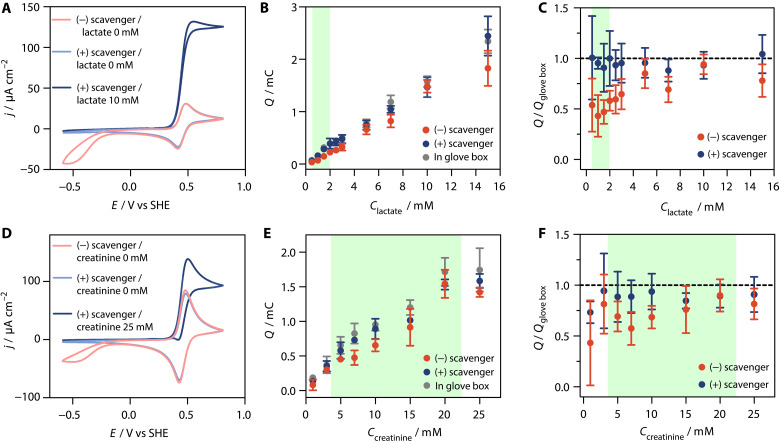
Lactate and creatinine biosensors. (**A**) CVs (2 mV s^−1^) of GCE modified with the Os complex–modified polymer and lactate oxidase in buffer (1 ml) containing CAT and AOx (pink trace), upon addition of paraformaldehyde (light blue trace), and upon addition of lactate (10 mM, dark blue trace). (**B**) Charge versus lactate concentrations in the presence of paraformaldehyde only (red circles), in the presence of all components of the scavenger (blue circles), and with all components of the scavenger under an inert atmosphere (gray circles). (**C**) Normalized charge versus lactate concentration. The charge measured under inert conditions was used for normalization. (**D**) CVs (2 mV s^−1^) of GCE modified with the Os complex–modified polymer and sarcosine oxidase in buffer (1 ml) containing creatinase, creatininase, AOx, and CAT (pink trace), with added EtOH (250 mM, light blue line) and with added creatinine (25 mM, dark blue line). (**E**) Charge versus creatinine concentrations in the absence of the scavenger (red circles), in the presence of all components of the scavenger (blue circles), and with all components of the scavenger under an inert atmosphere (gray circles). (**F**) Normalized charge versus creatinine concentration. The charge measured under inert conditions was used for normalization. The green area indicates the normal range for lactate (*61*) in blood and creatinine in urine (*62*). All samples consisted of phosphate buffer (pH 7.5, 100 mM) with substrate concentrations as indicated. All experiments were performed in ambient air, unless stated otherwise. For [(B), (C), (E), and (F)], the measurements were performed with the SPEs. The applied potential is 0.4 V versus the pseudo-RE in the SPE. Data are the average of *n* = 3 repetitions, and error bars show the standard deviation.

We first screened for potential cross-reactions between the scavenger system and the sensing enzymes immobilized on GCEs in the Os complex–modified hydrogel film using cyclic voltammetry. Similarly to GOx (fig. S5), the lactate oxidase–modified electrode did not generate any catalytic current in the presence of paraformaldehyde (Fig. 7A). Upon the addition of lactate, a catalytic wave was observed corresponding to lactate oxidation by lactate oxidase and electron mediation through the Os complex–modified polymer to the electrode (Fig. 7A). The catalytic current in the presence of paraformaldehyde declined with a half-life of at least 2 hours (fig. S20). This timescale is largely sufficient for point-of-use lactate sensing via coulometry, which typically occurs within 10 min. These results confirm the absence of unwanted cross-reactions, demonstrating the compatibility between the paraformaldehyde-based oxygen scavenger and the lactate-sensing electrode.

In the case of the creatinine sensor, one or several of the enzymes in the cascade for creatinine sensing (sarcosine oxidase, creatininase, and creatinase) are strongly inhibited by paraformaldehyde (fig. S21). To address this, we substituted paraformaldehyde with ethanol as the reducing agent for AOx. The ethanol-based O_2_ scavenging systems suppressed oxygen reduction currents, while adding creatinine generated the expected catalytic wave for analyte oxidation catalyzed by the three-enzyme cascade (Fig. 7D). This validates the absence of cross-reactions between the ethanol-based AOx scavenger and the enzymes for creatinine detection (sarcosine oxidase, creatinase, and creatininase), making it suitable for O_2_ removal in creatinine sensing.

Following the successful screening on GCEs for both lactate and creatinine, we integrated the sensing enzymes and corresponding oxygen scavenging system into the SPE systems, replicating the approach used for the glucose biosensors. This enabled the fabrication of single-use, self-filling lactate and creatinine biosensors. The effects of the O_2_ scavenging systems were tested across a broad range of analyte concentrations using chronocoulometry (Fig. 7). In the absence of the O_2_ scavenger, charges measured at 0.4 V versus the pseudo-RE after 10 min for the low millimolar concentration range were about 50% lower compared to those measured under an inert atmosphere for both sensors. Higher concentrations (>5 mM for lactate and >10 mM for creatinine) showed less charge loss, which is consistent with the results observed for glucose sensing.

When the O_2_ scavenger was present, the charge measured in air closely matched those measured in the glove box for both lactate and creatinine in the 0.5 to 15 mM range. This demonstrates the potential for interference-free and calibration-free lactate and creatinine sensing in point-of-use applications even at low substrate concentrations.

The possibility of using different reducing agents for AOx expands the potential for analyte diversification. Paraformaldehyde, used for glucose and lactate biosensors, is a practical choice for sensor fabrication because of its solid form. For biosensors inhibited by paraformaldehyde, such as the creatinine biosensor, alternative reducing agents such as ethanol can be used. This volatile alcohol can also be stabilized in a gelled form (*55*), releasing dissolved alcohol upon the addition of an aqueous sample.

### Storage stability

We conducted long-term stability tests on both the AOx-based O_2_ scavenger and oxidase-based biosensors integrated with the AOx-based O_2_ scavenger. The long-term stability of the AOx-based O_2_ scavenger was evaluated using cyclic voltammetry for the SPE stored in ambient air at 4°C (fig. S22). The O_2_ reduction current at −0.8 V versus the pseudo-RE was monitored to quantify the scavenging efficiency over time. The O_2_ reduction current obtained for sensors lacking the O_2_ scavenger was used for normalization. The AOx scavenging system, using paraformaldehyde as the reducing agent, exhibited effective O_2_ removal for 7 days, followed by the appearance and gradual increase in the O_2_ reduction current, indicating a decrease in AOx activity after 1 week of storage (fig. S22). To investigate whether this decrease resulted from paraformaldehyde, we substituted it with ethanol and repeated the storage stability test (fig. S23). The similar performance trend observed with ethanol suggests that paraformaldehyde does not significantly affect AOx stability. Further stabilization of AOx within the sensor strip may be achieved by optimizing the AOx deposition, which was performed through direct drop-casting of the AOx solution in the current study.

The storage stability of oxidase-based biosensors integrated with the AOx-based O_2_ scavenger was evaluated by monitoring the charge with a 10 mM substrate as a function of the storage time. For glucose and lactate biosensors, paraformaldehyde was used as a reducing agent for the AOx-based O_2_ scavenger, while ethanol was used for creatinine biosensors (fig. S24). The creatinine biosensors were stable for at least 8 days (fig. S24C), while the glucose and lactate biosensors showed a rapid decline in charge, losing their activity within 5 days (fig. S24, A and B). This suggests that paraformaldehyde releases formaldehyde vapor during storage, which inhibits the GOx and lactate oxidase activity. Storage of the sensor with the capillary channel exposed to humidity from air, as performed in this stability test, may be the cause for paraformaldehyde depolymerization (*37*). Optimization of the storage conditions through packaging with desiccants or using alcohols stabilized in a solid form (*55*) could offer a viable approach for developing storage-stable biosensors.

## DISCUSSION

We demonstrate that AOx, in combination with paraformaldehyde and catalase, enables rapid and complete oxygen removal from microliter-volume electrochemical biosensors. Like many oxidases, AOx is highly specific for its reducing substrate, namely short-chain primary alcohols and paraformaldehyde. Hence, AOx is suitable as a biocatalyst for O_2_ removal in biosensing of any analyte other than its specific substrates. Unlike most oxidases, AOx is highly specific for its reaction with O_2_, serving as an oxidizing substrate. This distinctive feature is critical for preventing AOx from donating electrons to the classical electron mediators, such as Fc(MeOH)_2_ and Os complex–modified polymers, used in electrochemical biosensing. Therefore, the AOx-based oxygen scavenging system does not interfere with the electron transfer pathway responsible for signal detection.

We validated the applicability of the AOx-based scavenger by constructing a GOx-based electrochemical biosensor that is insensitive to oxygen within the glucose concentration range relevant to diabetes management. Previously, oxygen interference could only be mitigated (*2*) by using alternative biorecognition elements, such as glucose dehydrogenases (*38*) and GOx variants with reduced reactivity to oxygen (*56*, *57*) or by specifically designing the immobilization matrix for GOx to limit the oxygen reduction reaction (*18*, *40*, *58*, *59*). However, these methods often entail compromises in robustness (*60*), sensitivity, selectivity (*38*), linear range, or complexity of the biosensor (*57*). GOx remains the most robust, highly specific, and easily produced biorecognition element for glucose detection (*38*). Its combination with the AOx scavenger effectively overcomes its primary limitation (its reaction with oxygen), thus making GOx an ideal sensing enzyme for electrochemical glucose biosensors. In addition, we demonstrate that the AOx-based scavenger, using either paraformaldehyde or ethanol as the reducing agent, is compatible with other oxidase-based biosensors, such as those for lactate and creatinine. These results demonstrate that the AOx-based biosensor can enable a broad range of oxidase-based biosensors that were previously impractical because of excessive oxygen sensitivity, especially those targeting low analyte concentrations.

## MATERIALS AND METHODS

### Materials

AOx solution from *P. pastoris*, AOx from *Candida boidinii*, AOx from *Hansenula polymorpha*, GOx from *Aspergillus niger*, catalase from bovine liver, and sarcosine oxidase from *Bacillus* sp. were purchased from Merck. Lactate oxidase from recombinant microorganism, creatininase from recombinant *Escherichia coli*, and creatinase from recombinant *E. coli* were purchased from Creative Enzymes. Unless otherwise specified, all reagents were purchased from commercial suppliers and used as received.

### Enzymatic assay for AOx

A solution of 2,2′-azino-bis(3-ethylbenzothiazoline-6-sulfonic acid) (2.8 ml, 1.0 mg ml^−1^ in phosphate buffer, 100 mM, pH 7.5) was mixed with a solution of peroxidase (10 μl, 250 U ml^−1^ in ultrapure water). The absorbance at 405 nm was monitored until it became constant. Then, the solution of substrate (0.1 ml in ultrapure water) was added and mixed. The absorbance at 405 nm was monitored until it became constant. Then, the solution of AOx (0.1 ml, 0.1 U ml^−1^) in cold phosphate buffer (100 mM, pH 7.5) was added and mixed. The increase in the absorbance at 405 nm over 5 min was used to calculate the reaction rate of the different substrates with the different AOx. The substrates were methanol, ethanol, propanol, butanol, pentanol, and formaldehyde. Each alcohol was tested with substrate concentrations of 0.5, 1, 2.5, 5, and 10 mM. Formaldehyde was tested with substrate concentrations of 0.1, 0.3, 0.5, 0.7, 0.9, 2, 4, 7, 10, 15, 30, and 50 mM. For determining the relative reaction rates of methanol, ethanol, formaldehyde, glucose, lactate, sarcosine, and creatinine, their concentration was 10 mM. Aqueous formaldehyde was prepared by heating paraformaldehyde in pure water at 60°C overnight. The formaldehyde concentration of the resulting clear solution was determined by Karl Fischer titration. A stock solution of paraformaldehyde (5 mg ml^−1^) was prepared, filtered with a polytetrafluoroethylene syringe filter (0.45 μm, 13 mm), and used for the enzymatic assay.

### Microelectrode measurements

A carbon microelectrode (11 μm in diameter) was used as the working electrode to detect the O_2_ reduction current with chronoamperometry. A Pt wire was used as the counter electrode and Ag/AgCl (3 M KCl) as the RE. A potential at −0.8 V versus Ag/AgCl was applied. The O_2_ reduction current at the carbon microelectrode was calibrated on the basis of the O_2_ concentration measured with an optical O_2_ meter (Pyro Science: FireStingO2). The O_2_ meter is too slow to follow the rapid change in O_2_ concentration but provides an accurate O_2_ concentration at equilibrium. The advantage of the carbon microelectrode is its rapid response time, and upon calibration with the fluorescence-based O_2_ sensor, it enables accurate monitoring of the changes in O_2_ concentration. The calibration of the microelectrode with the optical O_2_ sensor was performed for 60 s in phosphate buffer (1 ml, 100 mM, pH 7.2) containing catalase (2000 U ml^−1^) and different concentrations of paraformaldehyde. After 60 s, AOx (10 U ml^−1^) was added, the solution was stirred for 10 s, and the current for O_2_ reduction was then recorded to monitor the O_2_ concentration over time.

### Polymer synthesis

#### Poly(1-vinylimidazole)

1-Vinylimidazole (6 ml) and azobisisobutyronitrile (120 mg) were polymerized at 70°C for 2 hours. After allowing the reaction mixture to cool, the resulting precipitate was dissolved in 50 ml of methanol at 50°C and left to stir overnight. The polymer solution was then added, under stirring, to a mixture of 800 ml of acetone and 200 ml of diethyl ether. The white precipitate that formed was filtered and dried under vacuum, yielding the polymer as a pale-yellow solid.

#### Poly(1-vinylimidazole)-Os(bpy)_2_Cl

See (*43*). K_2_OsCl_6_ (0.95 g, 2 mmol) and 2,2′-bipyridine (0.65 g, 4.2 mmol) were refluxed in dimethylformamide (20 ml) for 1 hour. After cooling the reaction mixture to room temperature (RT), it was filtered and washed with 10 ml of ethanol. The resulting filtrate was slowly added dropwise to 250 ml of diethyl ether under continuous stirring, which resulted in the formation of *cis*-bis(2,2′-bipyridine-*N*,*N*′)dichloroosmium(III). The solid product was collected by filtration, washed with diethyl ether, and air dried overnight. The complex (1.14 g) was then dissolved in a mixture of 23 ml of dimethylformamide and 11.5 ml of methanol. Subsequently, 100 ml of a 1% (w/w) aqueous solution of sodium dithionite was added dropwise to the stirred solution. After storing the solution overnight at 4°C, the product [*cis*-bis(2,2′-bipyridine-*N*,*N*′)dichloroosmium(II)] formed as a purple solid. This solid was collected by filtration and washed sequentially with water, methanol, and diethyl ether until the filtrates were clear. Last, poly(1-vinylimidazole) and *cis*-bis(2,2′-bipyridine-*N*,*N*′)dichloroosmium(II) were dissolved in ethanol in a 3:1 molar ratio and refluxed for 3 days. The reaction mixture was then filtered, and the polymer was precipitated by adding the polymer solution to a stirred mixture of 800 ml of acetone and 200 ml of diethyl ether (80:20, v/v). The solid product was filtered and dried under vacuum.

### Electrode preparation

GCEs were sequentially polished using Al_2_O_3_ powder slurries with particle sizes of 1, 0.5, and 0.05 μm, followed by thorough rinsing with distilled water and sonication in 50% ethanol for 20 min. The electrodes were subsequently dried using a stream of nitrogen gas.

#### GCE for glucose sensing

The Os complex–modified polymer (50 wt %), GOx (40 wt %), and poly(ethylene glycol) diglycidyl ether (PEGDGE) (10 wt %) as a cross-linker were dissolved in potassium phosphate buffer (100 mM, pH 8.1) and drop-cast on the GCE at a total loading of 200 μg cm^−2^. The electrodes were dried overnight at 4°C and left for 40 min at RT before they were used. The O_2_ scavenging system was AOx (10 U ml^−1^), CAT (2000 U ml^−1^), and paraformaldehyde (5 mg ml^−1^) dissolved in the electrolyte for the measurement with the modified GCEs.

#### GCE for lactate sensing

The Os complex–modified polymer (40 wt %), lactate oxidase (50 wt %), and PEGDGE (10 wt %) were dissolved in potassium phosphate buffer (100 mM, pH 8.1) and drop-cast on the GCE at a total loading of 500 μg cm^−2^. The electrodes were dried overnight at 4°C and left for 40 min at RT before they were used. The O_2_ scavenging system was AOx (10 U ml^−1^), CAT (2000 U ml^−1^), and paraformaldehyde (5 mg ml^−1^) dissolved in the electrolyte for the measurement with the modified GCEs.

#### GCE for creatinine sensing

The Os complex–modified polymer (46 wt %), sarcosine oxidase (46 wt %), and PEGDGE (8 wt %) were dissolved in potassium phosphate buffer (100 mM, pH 8.1) and drop-cast on the GCE at a total loading of 500 μg cm^−2^. The electrodes were dried overnight at 4°C and left for 40 min at ambient temperature before they were used. The measurements were performed in phosphate buffer containing creatinine (25 mM), creatininase (60 U ml^−1^), creatinase (18 U ml^−1^), and the O_2_ scavenging system consisting of AOx (10 U ml^−1^), CAT (2000 U ml^−1^), and EtOH (50 mM).

### Screen-printed electrode (SPEs)

SPEs (Dropsens, Metrohm) were used directly without any additional cleaning or polishing.

#### SPEs for glucose sensing

The Os complex–modified polymer (50 wt %), GOx (40 wt %), and PEGDGE (10 wt %) were dissolved in phosphate buffer (100 mM, pH 8.1) and drop-cast on the working electrode at a total loading of 200 μg cm^−2^. The electrodes were allowed to dry overnight at 4°C. Before measurements, the electrodes were equilibrated at RT for 40 min. AOX (40 U ml^−1^) and CAT (4000 U ml^−1^) in Milli-Q water (5 μl) were drop-cast on thin paper (Japico), which was placed on a Teflon surface and left to dry. Paraformaldehyde was scattered by a fine sieve in its solid form on the dried thin paper (corresponding to ~50 mg ml^−1^ upon filling the SPEs). AOx (40 U ml^−1^) and CAT (4000 U ml^−1^) in Milli-Q water (10 μl) were drop-cast on the inside of the top cover of the capillary and allowed to dry for 30 min.

#### SPEs for lactate sensing

The Os complex–modified polymer (50 wt %), lactate oxidase (50 wt %), and PEGDGE (10 wt %) were dissolved in phosphate buffer (100 mM, pH 8.1) and drop-cast on the working electrodes at a total loading of 50 μg cm^−2^. After modification, the electrodes were allowed to dry overnight at 10°C and 80% humidity. Before measurements, the electrodes were equilibrated at RT for 40 min. AOx (40 U ml^−1^) and CAT (4000 U ml^−1^) in Milli-Q water (5 μl) were drop-cast on thin paper (Japico), which was placed on a Teflon surface and left to dry. Paraformaldehyde was scattered by a fine sieve in its solid form on the dried thin paper (corresponding to ~50 mg ml^−1^ upon filling the SPEs). AOx (40 U ml^−1^) and CAT (4000 U ml^−1^) in Milli-Q water (10 μl) were drop-cast on the inside of the top cover of the capillary and allowed to dry for 30 min.

#### SPEs for creatinine sensing

The Os complex–modified polymer (46 wt %), sarcosine oxidase (46 wt %), and PEGDGE (8 wt %) were dissolved in phosphate buffer (100 mM, pH 8.1) and drop-cast on the working electrodes at a total loading of 50 μg cm^−2^. After modification, the electrodes were allowed to dry overnight at 10°C and 80% humidity. Before measurements, the electrodes were equilibrated at RT for 40 min. AOx (40 U ml^−1^), creatininase (2 mg ml^−1^), and creatinase (4 mg ml^−1^) in Milli-Q water (5 μl) were deposited onto thin paper (Japico), which was placed on a Teflon surface and left to dry. AOx (40 U ml^−1^) and CAT (4000 U ml^−1^) in Milli-Q water (10 μl) were drop-cast on the inside of the top cover of the capillary and allowed to dry for 30 min. Ethanol (250 mM) was dissolved in the sample.

### Blood sample analysis

Blood was purchased from Dunn Lab, where it was collected from a single human donor. The blood was only treated with sodium citrate as an anticoagulant. Specific glucose concentrations (3, 5, 7, 10, and 15 mM) were added to the blood following the internal standard addition method.

## Supplementary Materials

### This PDF file includes:

Supplementary text Figs. S1 to S24 Tables S1 to S4 References
