# Supplementary material for: A universal oxygen scavenger for oxidase-based biosensors
Source: Sci Adv. 2025 Sep 12;11(37):eadw6133. doi: 10.1126/sciadv.adw6133 (PMC12429024; doi:10.1126/sciadv.adw6133)
Supplement: Supplementary file 1 — Supplementary text Figs. S1 to S24 Tables S1 to S4 References [file sciadv.adw6133_sm.pdf]

Science Advances
AAAS

Supplementary Materials for

A universal oxygen scavenger for oxidase-based biosensors

Huijie Zhang *et al.*

Corresponding author: Nicolas Plumeré, nicolas.plumere@tum.de

Sci. Adv. **11**, eadw6133 (2025)
DOI: 10.1126/sciadv.adw6133

This PDF file includes:

Supplementary text
Figs. S1 to S24
Tables S1 to S4
References

Supplementary text

Mathematical modeling and simulation of the oxygen scavenging system

3D sensor geometry

The sensor is a thin and narrow strip with total dimensions of 8 mm in length (x direction), 3 mm in depth (y direction), and 50 µm in height (z direction). The capillary opening is exposed to air; all other sides of the device are closed (**Fig. S11**).

Reduction to 2D-symmetry along the XY plane

Symmetry in the z plane is assumed, resulting in the reduction of the problem from a 3D problem to a 2D problem. A cross-section of the sensor is shown by taking a slice in the xy plane. The sensor has a counter, reference, and working electrodes. The working electrode is furthest away from the opening; the left edge of this electrode is taken to be the limit that the oxygen front can travel before sensor performance is affected (**Fig. S12**).

Reduction to 1D-symmetry along the X axis

Symmetry along the x-axis is assumed, allowing for a further reduction of the problem from a 2D problem to a 1D problem. The resulting geometry of the system is shown by a line along the x-axis (**Fig. S13**).

Reaction schematic for the AOx-based oxygen scavenging system

We start with the complete schematic shown in the main text. In the same way that the geometry was able to be simplified by the introduction of assumptions, the reaction scheme used for simulations was also simplified (**Fig. S14**).

Rate Limiting Step

Paraformaldehyde $\xrightarrow{H_2O}$ formaldehyde (Reaction 1)

AOx reaction: O_2 +formaldehyde + H_2O $\xrightarrow{AOx}$ formic acid + H_2O_2 (Reaction 2)

CAT reaction: $H_2O_2 \rightarrow H_2O + 1/2\ O_2$ (Reaction 3)

Net reaction: $O_2 + 2\ CH_2O \rightarrow 2\ HCOOH$ (Reaction 4)

The depolymerization of paraformaldehyde (reaction 1) and the disproportionation of hydrogen peroxide (reaction 3) are assumed to be much faster than the enzymatic reaction between formaldehyde and oxygen (reaction 2). Therefore, reaction 2 is assumed to be the rate determining step, such that the rate of oxygen removal in the device can be modeled by considering this reaction only.

Final Reaction Scheme

Introduction of this assumption along with consideration that an additional half molecule of oxygen (reaction 3) is consumed in the overall process results in the final reaction scheme (**Fig. S15).** Because of the assumption of a fast and complete conversion of paraformaldehyde to formaldehyde, the initial concentration of formaldehyde is taken as the initial concentration of paraformaldehyde.

Reaction Schematic for ethanol scavenging system

The reaction scheme for ethanol is the same as for the formaldehyde system with the only difference that there is no precursor reaction. (reactions 5-7) The same rate limiting step assumption for the hydrogen peroxide conversion applies resulting in the simplified reaction scheme shown in **Fig. S16**.

AOx reaction: $O_2 + RCH_2OH \xrightarrow{AOx} RCHO + H_2O_2$ (Reaction 5)
CAT reaction: $H_2O_2 \rightarrow H_2O + 1/2\ O_2$ (Reaction 6)
Net reaction: $O_2 + 2\ RCH_2OH \rightarrow 2\ RCHO + 2H_2O$ (Reaction 7)

System description

Fast filling of the sensor

The process of filling the sensor by means of capillary action is assumed to be fast, such that the fluid domain for the sensor can be assumed to be full and stationary at the initial condition.

Reactions in the sensor with a constant influx of oxygen from the capillary opening

The oxygen scavenging reactions catalyzed by AOx by means of a ternary complex mechanism begin immediately when the capillary is filled, before the application of the electrode potential. The important feature of this mechanism is the two Michaelis-Menten kinetic constants, one for the substrate (K_S), which can be either formaldehyde or ethanol, and another for oxygen (K_o).
Due to the opening of the capillary, there will be a constant influx of oxygen. We will assume that the concentration of oxygen at the capillary opening will remain constant and equal to the bulk value of oxygen in the air.
It will also be assumed that oxygen will flow into the capillary without any limitations due to the geometry. This assumption was relevant for the reduction of the problem from a 2D problem in the xy-plane to a 1D problem along the x-axis. As a result, the height of the sensor is not needed as a parameter in the simulation.

Governing Equations

Material balances on oxygen (O) and on the substrate (S) in the sensor volume result in the two main governing equations that define the time-dependent concentrations of oxygen and the substrate (formaldehyde or ethanol) in the sensor.

$$\frac{\partial C_O}{\partial t} = D_O \frac{\partial^2 C_O}{\partial x^2} - \frac{k_{cat} C_S C_O C_E}{K_S C_O + K_S C_O + C_S C_O} \qquad \text{(Eq. S1)}$$

$$\frac{\partial C_S}{\partial t} = D_S \frac{\partial^2 C_S}{\partial x^2} - \frac{2 k_{cat} C_S C_O C_E}{K_S C_O + K_S C_O + C_S C_O} \qquad \text{(Eq. S2)}$$

The rate expression for the enzymatic catalysis in the capillary is based on a biproduct-bisubstrate Ping-Pong mechanism, which is in terms of the kinetic model, analogous to the ternary complex mechanism (despite the two mechanisms differ in the sequence of the individual steps) (*63*). We will consider formaldehyde or ethanol being oxidized by alcohol oxidase in the presence of oxygen. The factor of 2 in the rate expression for the substrate reflects the 1:2 stoichiometric ratio between O_2 and the substrate in the net reaction.

Boundary Conditions

Oxygen

The capillary opening (at x=0) is exposed to oxygen, such that the concentration of oxygen at the inlet is set to a specified value (Dirichlet boundary condition), the bulk concentration of oxygen in the surrounding atmosphere.

$$(C_O)_{x=0} = C_O^{Bulk} \qquad \text{(Eq. S3)}$$

The right side of the sensor (at x=l) is closed such that it is not possible for oxygen to diffuse out of the sensor. This results in a constraint on the value of the flux (Neumann boundary condition). As a result, the flux of oxygen leaving the sensor is equal to zero.

$$\left(\frac{\partial C_O}{\partial x}\right)_{x=l} = 0 \qquad \text{(Eq. S4)}$$

Substrate

The substrate is confined to the capillary of the sensor, and so the flux at both capillary boundaries (Neumann boundary condition) is taken to be zero.

$$\left(\frac{\partial C_S}{\partial x}\right)_{x=0} = \left(\frac{\partial C_S}{\partial x}\right)_{x=l} = 0 \qquad \text{(Eq. S5)}$$

Initial Conditions

The capillary is assumed to be filled very quickly. As a result, it is assumed that the system starts as a stationary fluid in which the oxygen is uniformly at its maximum value (concentration of dissolved oxygen in an aqueous solution exposed to ambient air), and in which the formaldehyde is uniformly at its nominal value.

Scaling and Solving

The system of PDE equations was solved numerically using MATLAB. In particular, the pdepe solver was used, which makes use of the Finite Difference method.

Before solving, the main variables of concentration, time, and space were scaled with respect to their maximum values. The primary purpose of scaling in this instance was to maximize the stability of results obtained by the solver.

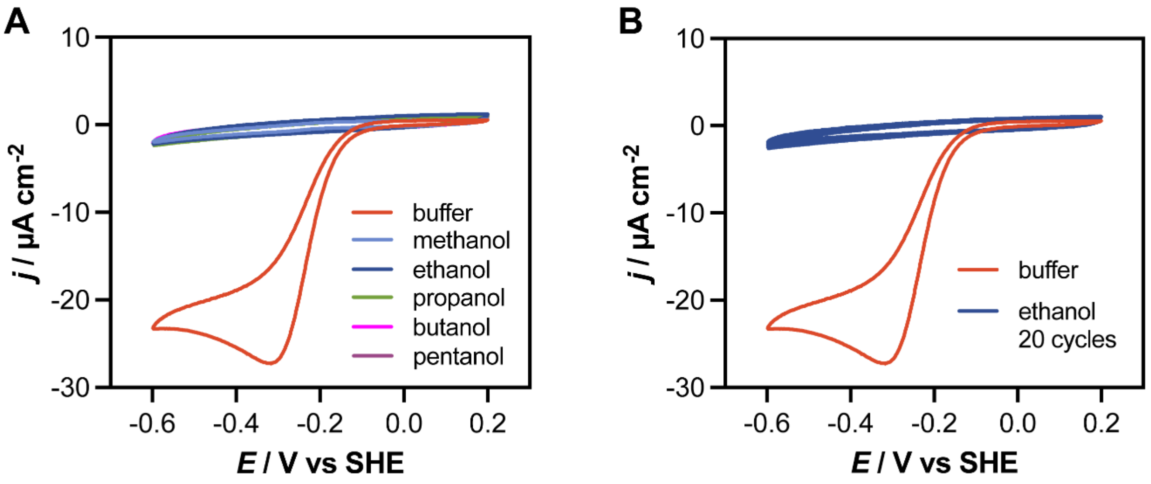

Fig. S1. Cyclic voltammograms (CV) of the oxygen scavenger in solution. (A) CVs (2 mV s^{-1}) at GCE in unstirred electrolyte (1 mL) (red line) and upon addition of AOx, CAT with different alcohols as substrates. **(B)** CVs (2 mV s^{-1}) at GCE in unstirred electrolyte (1 mL) (red line) and 20 successive CVs upon addition of AOx, CAT and ethanol as O_2 scavenger (blue lines). All experiments were performed in phosphate buffer (100 mM, pH 7.5), Alcohols: 50 mM, AOx: 10 U mL^{-1}, CAT: 2000 U mL^{-1}.

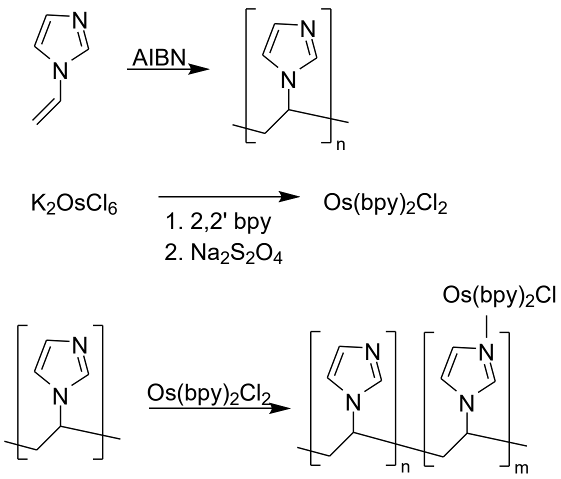

1

2

3

Fig. S2. Polymer synthesis. Synthesis of Poly(1-vinylimidazole)-Os(bpy)$_2$Cl)

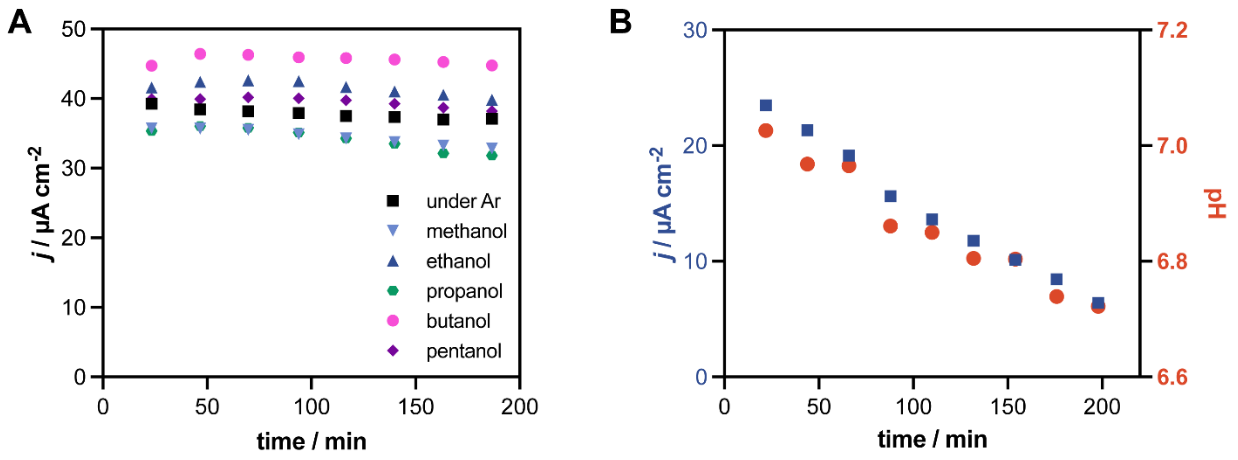

Fig. S3. The effect of O_2 scavenger products on GOx using different alcohols and paraformaldehyde as reducing agents. (A) Catalytic current for glucose oxidation over time in phosphate buffer (100 mM, pH 7.5, 1 mL) containing GOx（15 U mL^{-1}）and glucose (50 mM) under Ar and with O_2 scavenger using different alcohols as substrates. The oxygen scavenger contains the alcohol (50 mM), AOx (10 U mL^{-1}), and CAT (2000 U mL^{-1}). **(B)** Catalytic current for glucose oxidation and pH changes over time in phosphate buffer (100 mM, pH 7.2, 1 mL) containing GOx（15 U mL^{-1}）and glucose (50 mM). The O_2 scavenger contains paraformaldehyde (5 mg mL^{-1}), AOx (10 U mL^{-1}), and CAT (2000 U mL^{-1}). For all experiments $Fc(MeOH)_2$ (100 μM) was used as an electron mediator. The solutions were stirred for 30 s between each measurement. The catalytic current was measured from the CVs at a potential of 0.8 V *vs* SHE. GCEs were used as working electrodes for all experiments.

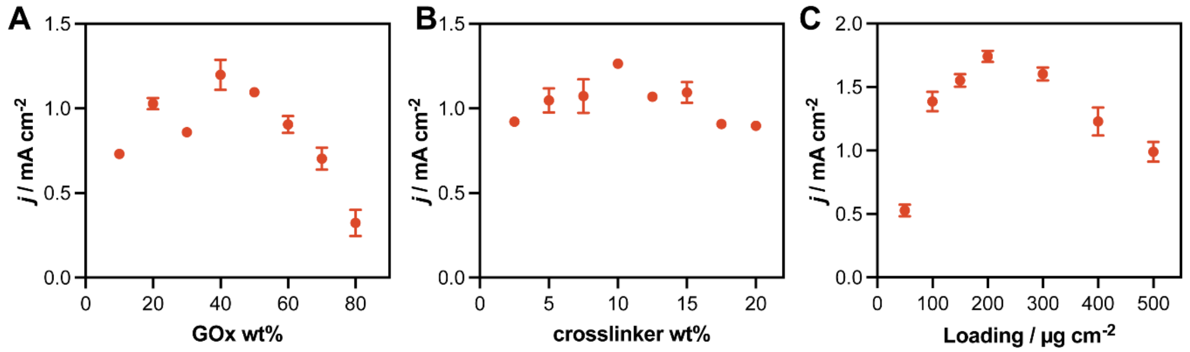

Fig. S4. Optimization of the composition of the Os-complex-modified film containing GOx. **(A)** Dependency of the GOx wt% on catalytic current density with total loading mass 100 µg cm^{-2} and crosslinked with 10wt% PEGDGE. **(B)** Dependency of the cross-linker wt% on catalytic current density with total loading mass 100 µg cm^{-2}, the film contains 40 wt% GOx. **(C)** Dependency of the loading mass (film thickness) on catalytic current density. The film contains Os-complex modified polymer (50 wt%), GOx (40 wt%), and PEGDGE (10 wt%). All experiments were performed with GCEs using 50 mM glucose in phosphate buffer (50 mM, pH 7.5). Under Ar purging. The catalytic current was measured from the CVs at a potential of 0.8 V *vs* SHE. Data are the average of n = 3 datasets and error bars show standard deviation.

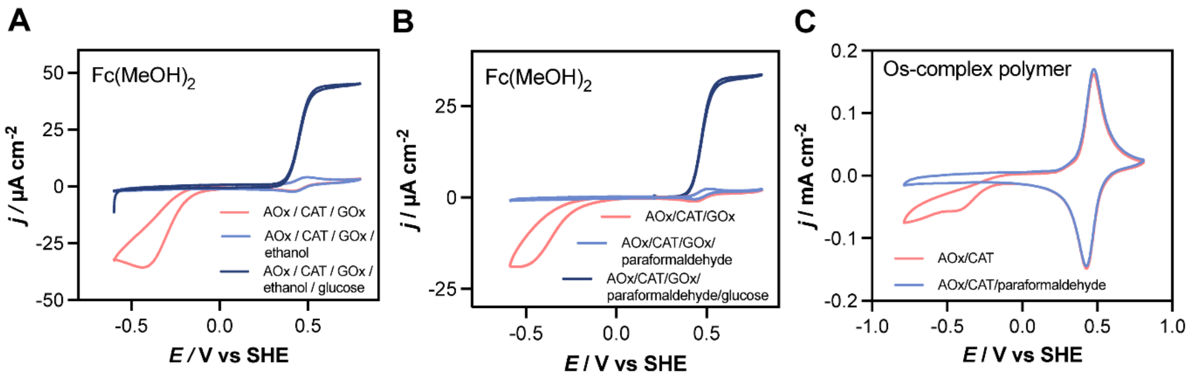

Fig. S5. **AOx-based O_2 removal for glucose biosensing. (A)** CVs (2 mV s^{-1}) at GCE in electrolyte (1 mL) contains AOx, CAT, $Fc(MeOH)_2$ and GOx (light red line), upon addition of ethanol (light blue line), and upon addition of glucose (dark blue line). **(B)** CVs (2 mV s^{-1}) at GCE electrolyte (1 mL) contains AOx, CAT, $Fc(MeOH)_2$ and GOx (light red line), upon addition of paraformaldehyde (light blue line), and upon addition of glucose (dark blue line). All experiments were performed in phosphate buffer (100 mM, pH 7.5), $Fc(MeOH)_2$: 100 µM, GOx: 15 U mL^{-1}, EtOH: 50 mM, AOx: 10 U mL^{-1}, CAT: 2000 U mL^{-1}, glucose: 50 mM. **(C)** CVs (10 mV s^{-1}) of a modified GCE with Os-complex polymer (50 wt%), GOx (40 wt%) and PEGDGE (10 wt%) with total loading 200 µg cm^{-2} in electrolyte (1 mL) contains AOx and CAT (red line) and with addition of paraformaldehyde (blue line). All experiments were performed in phosphate buffer (pH 7.5, 100 mM), AOx: 10 U mL^{-1}, CAT: 2000 U mL^{-1}, paraformaldehyde: 5 mg mL^{-1}.

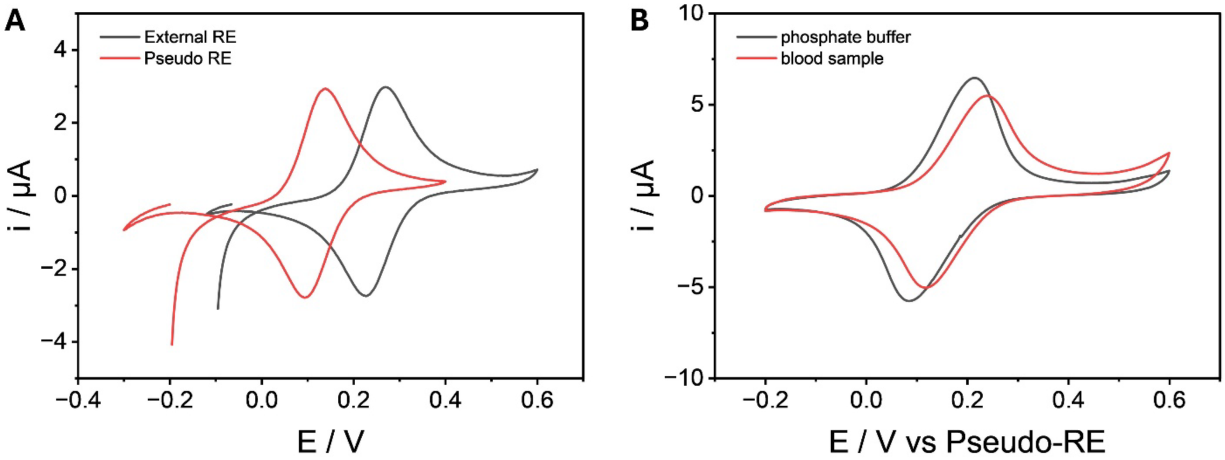

Fig. S6. Potential of the screen-printed pseudo reference electrode. (A) CVs of Os-complex modified polymer on SPE with capillary channel filled with phosphate buffer (pH 7.5, 100 mM) using an external Ag/AgCl (3M KCl) reference electrode (black trace), and using its internal Ag/AgCl paste Pseudo-reference electrode (red trace). **(B)** CVs of Os-complex modified polymer on SPE with a capillary channel filled with phosphate buffer (pH 7.5, 100 mM) (black trace) and filled with blood sample (red trace) using the internal Ag/AgCl paste Pseudo-reference electrode.

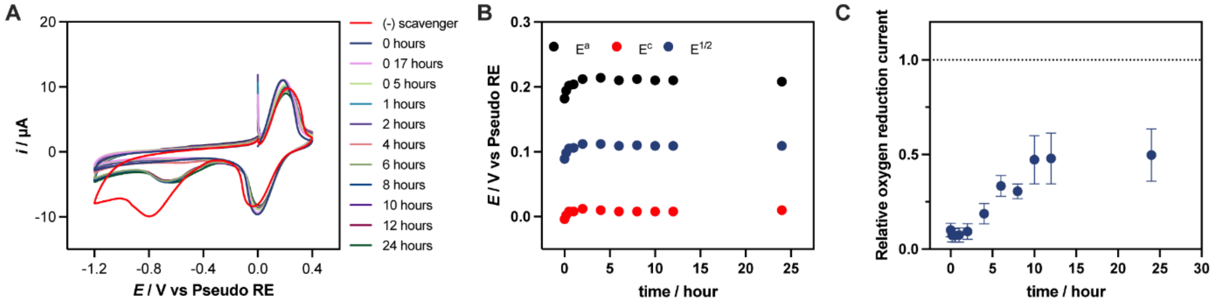

Fig. S7. Duration of O_2 scavenging in screen-printed electrodes exposed to air. (A) CVs of SPEs modified with Os-complex modified polymer in the presence of AOx / CAT / paraformaldehyde. All experiments were performed in phosphate buffer (pH 7.5, 100 mM). The O_2 scavenger contains paraformaldehyde (50 mg mL^{-1}), AOx (10 U mL^{-1}), and CAT (2000 U mL$^{-1)}$. SPEs modified with Os-complex modified polymer (50 wt%), GOx (40 wt%) and PEGDGE (10 wt%) with a total loading of 50 µg cm^{-2}. **(B)** Anodic (black dots) and cathodic (red dots) peak potentials, and the resulting $E_{1/2}$ values (blue dots) of the Os-complex to assess the stability of the potential of the Pseudo RE over time. **(C)** Current at -0.8 V vs. Pseudo RE extracted from (A). Current at 0 h in the absence of a scavenger was used for normalisation.

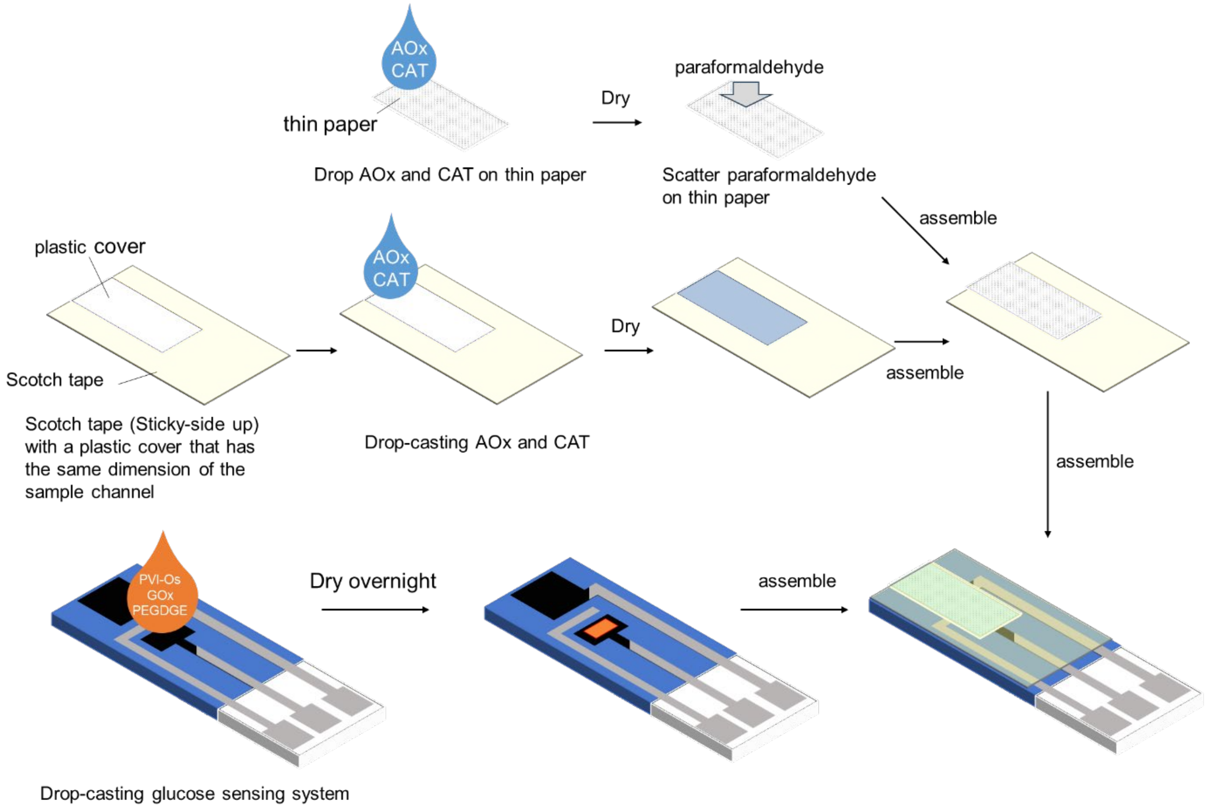

Fig. S8. Screen-printed electrode assembly. Assembly of a glucose biosensor with an oxygen scavenger system in a screen-printed electrode (SPE).

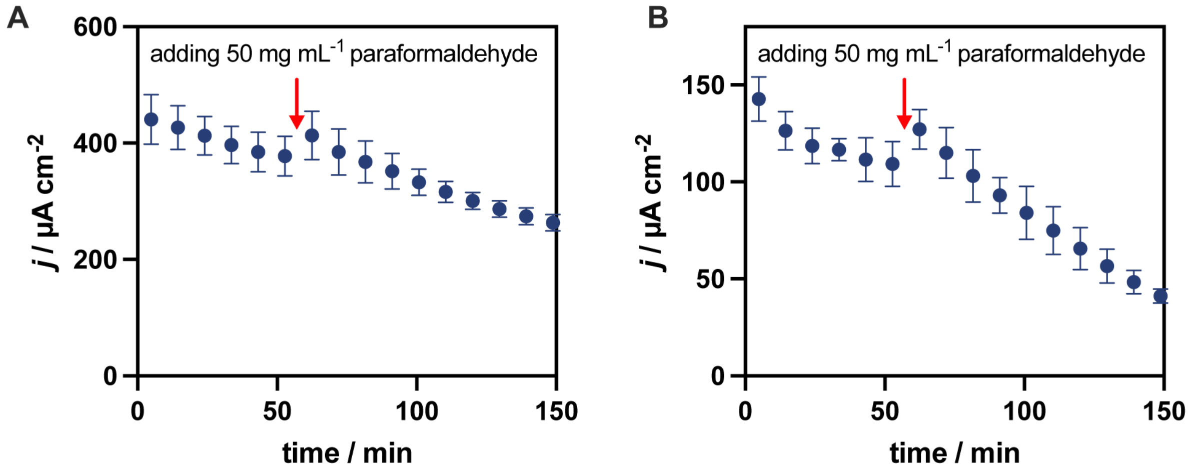

Fig. S9. The impact of paraformaldehyde on the activity of GOx and lactate oxidase. (A) Catalytic current density for glucose oxidation over time in the presence of an O_2 scavenger in electrolyte (1 mL) with 50 mM glucose. O_2 scavenger contains paraformaldehyde (50 mg mL^{-1}), AOx (10 U mL^{-1}), and CAT (2000 U mL$^{-1)}$. Paraformaldehyde was added at 1 h. GCE modified with Os-complex modified polymer (50 wt%), GOx (40 wt%) and PEGDGE (10 wt%) at a total loading of 200 µg cm^{-2}. **(B)** Catalytic current density for lactate oxidation over time in the presence of an O_2 scavenger in electrolyte (1 mL) with 10 mM lactate. All experiments were performed in phosphate buffer (pH 7.5, 100 mM). The scavenger contains paraformaldehyde (50 mg mL^{-1}), AOx (10 U mL^{-1}), and CAT (2000 U mL^{-1}). Paraformaldehyde was added at 1 h. GCE was modified with lactate oxidase (50 wt%), Os-complex modified polymer (40 wt%), PEGDGE (10 wt%) at a total loading of 500 µg cm^{-2}.

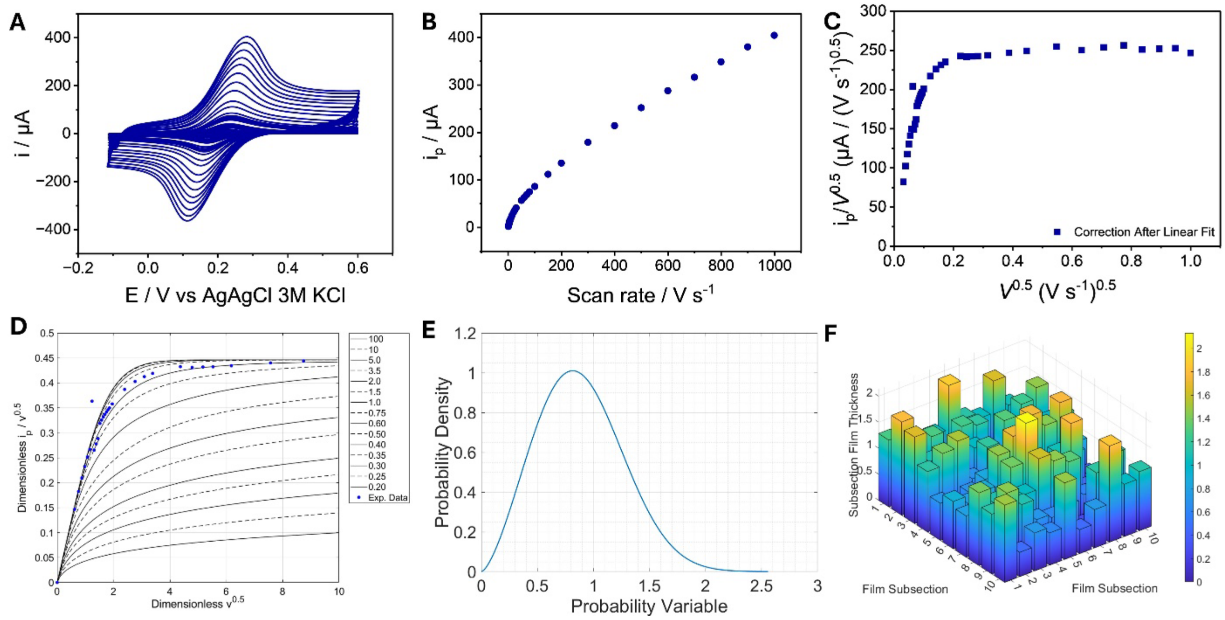

Fig. S10. Determination of the film thickness distribution by means of cyclic voltammetry (CV) (A) CVs of SPE modified with Os-complex modified polymer (50 wt%), GOx (40 wt%) and PEGDGE (10 wt%) at a total loading 200 µg cm^{-2}. Scan rate was increased from 1 mV s^{-1} to 1 V s^{-1}. **(B)** Anodic peak current (i_p) *vs* the corresponding scan rate (v). **(C)** Normalized peak current $i_p/v^{0.5}$ *vs* the square root of the scan rate $v^{0.5}$. Capacitance contributions to the CVs were corrected in the secondary plot in two steps. Firstly, a baseline correction of the entire data set was performed based on the i_p values of the CVs in the high scan rate range. Secondly, a linear fit of the peak currents of CVs with the lowest scan rates was performed. The y-intercept value was used as a baseline to correct all i_p values. **(D)** Dimensionless secondary plot with dimensionless $i_p/v^{0.5}$ vs dimensionless $v^{0.5}$, obtained from the data in c) using the data processing method as described in (*64*) that uses the linear and plateau regions directly to transform the dimensional secondary plot into its dimensionless form. The normalized peak current value at the characteristic dimensionless $w^{0.5}$ value (0.36) corresponds to a Weibull Distribution shape factor value of 2.5 when using the correlation as described in (*64*). **(E)** Resulting probability distribution function for a Weibull Distribution with shape factor equal to 2.5, which has a relative standard deviation of 43%. **(F)** Three-dimensional representation of a film with this shape factor for a random sampling of 100 points as a 10×10 grid. The y-axis represents the fraction of the thickness of the subsection relative to the average thickness of the film as a whole.

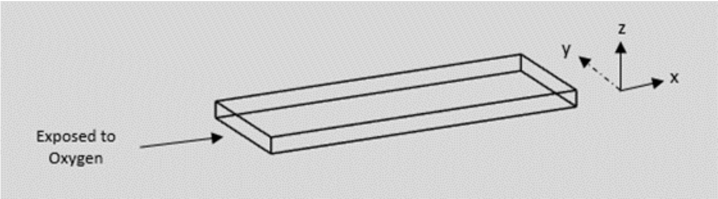

Fig. S11. 3D capillary geometry. The capillary opening of the sensor is continually exposed to oxygen.

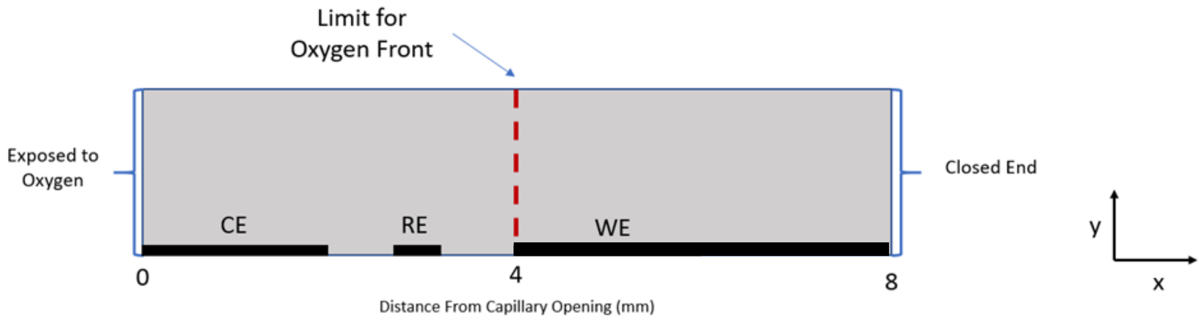

Fig. S12. 2D sensor geometry. The dimensionality was reduced from 3D to 2D by the identification of a symmetry plane.

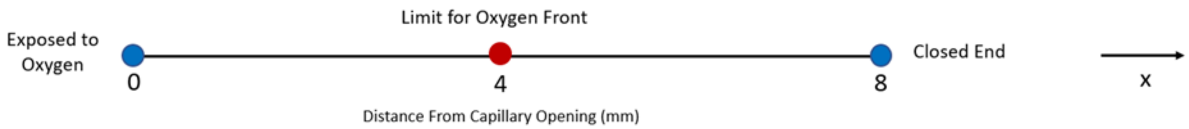

Fig. S13. 1D model of the sensor geometry. The electrodes appear as points. Further reduction of the model from 2D to 1D was possible by the identification of a second symmetry plane.

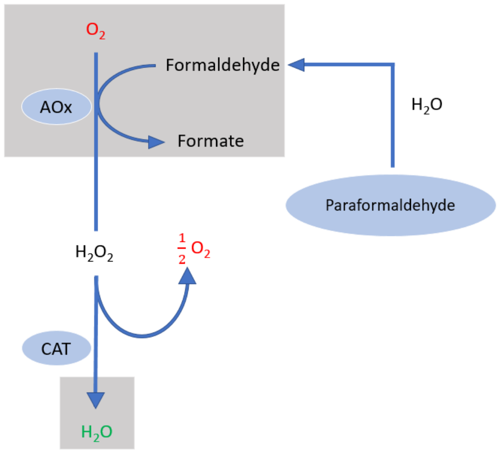

Fig. S14. Complete reaction scheme of the oxygen scavenging system. The unshaded sections are assumed to be extremely fast relative to the reactions in the shaded sections.

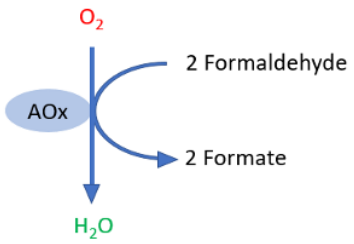

Fig. S15. Simplified reaction scheme with a focus on the rate limiting step of the overall oxygen scavenging process. The removal of oxygen by alcohol oxidase uses formaldehyde as the substrate via a ternary complex mechanism in which oxygen is the electron acceptor.

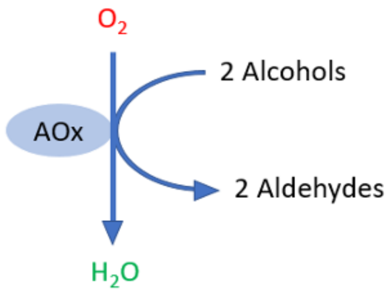

Fig. S16. Simplified reaction scheme of the oxygen scavenging process in which alcohol is used as the substrate. The reaction of alcohol oxidase is via a ternary complex mechanism in which oxygen is the electron acceptor.

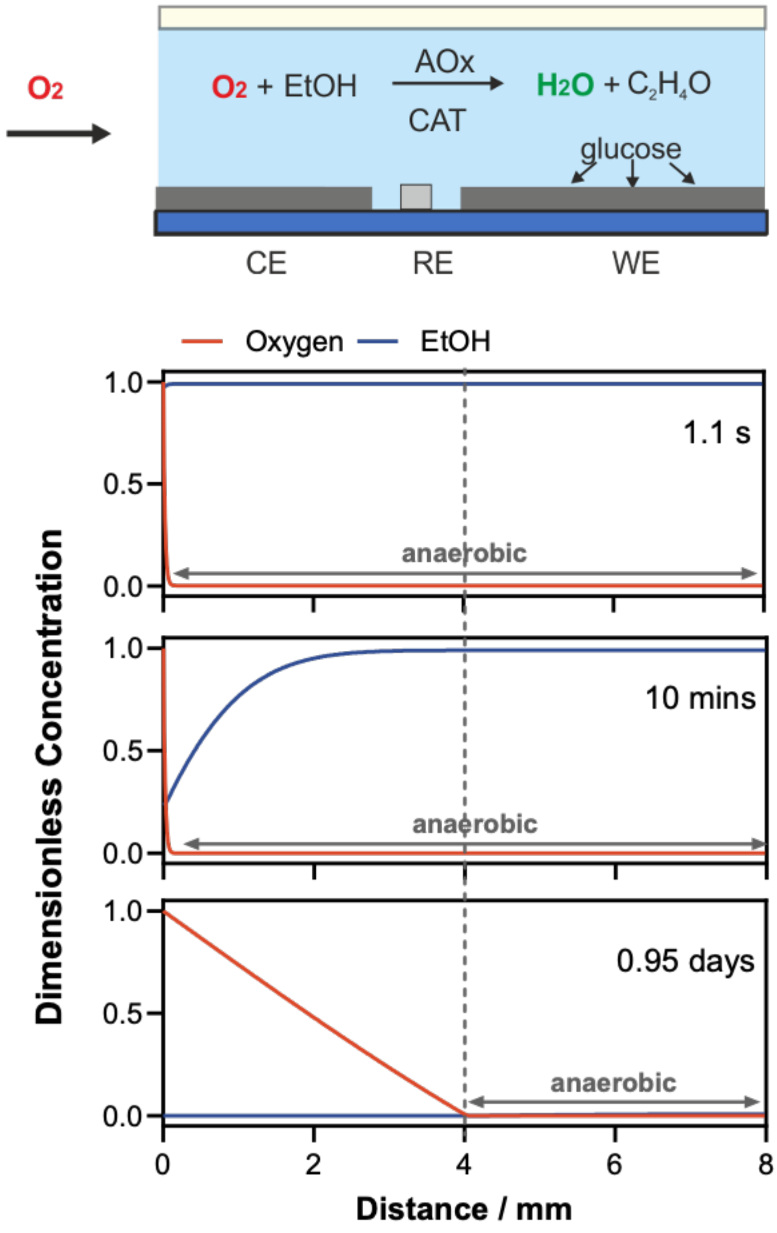

Fig. S17. **Simulations of the O_2 concentration and ethanol concentration within the capillary channel when using ethanol as a reducing agent.** The O_2 concentration and EtOH concentration within the channel of SPEs from simulations of the O_2 scavenging using EtOH as a reducing agent. The O_2 concentrations were simulated after depletion of the O_2 initially present in the sample (1.1 s), after 10 mins, and after 0.95 days when the O_2 reaches the sensing electrode. The simulations account for the continuous supply of O_2 through the capillary opening exposed to ambient air. Parameter values for the simulation are in Table S3.

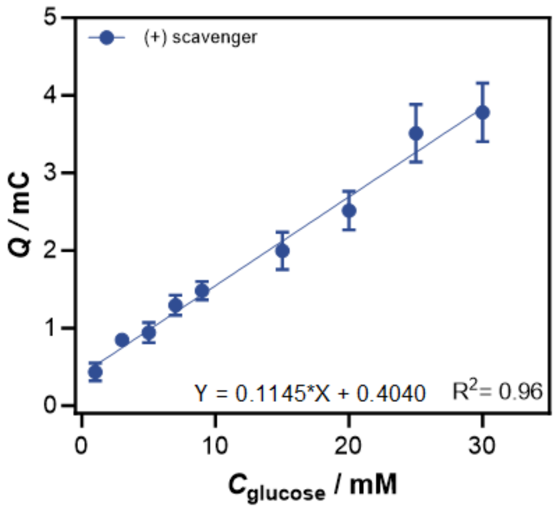

Fig. S18. Calibration curve of the glucose biosensor with O_2 scavenger in SPEs. Data correspond to those measured with the O_2 scavenger shown in **Fig. 5C.** The sensitivity of the biosensor obtained from the fitting is 114.5 µC / mM. The charge value of the blank (0 mM glucose) was determined to be 196.07 ± 27.45 µC. The limit of detection (LOD) of the biosensor, calculated as three times the standard deviation of the blank divided by the slope of the calibration curve, is determined to be 0.72 mM.

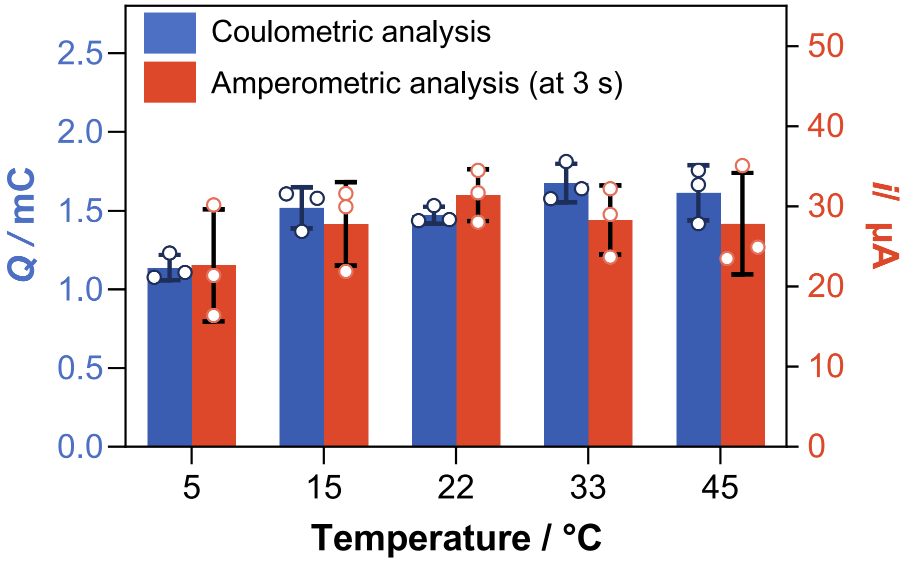

Fig. S19. Chronocoulometric analysis vs amperometric analysis (at 3 s) of SPEs at different Temperatures (5, 15, 22, 33, 45 °C) under ambient air. The SPEs were modified with Os-complex modified polymer (50wt%), GOx (40 wt%), and PEGDGE (10 wt%) with a total loading 200 μg cm^{-2}. The O_2 scavenger was incorporated (with scavenger system AOx / CAT / paraformaldehyde) into the SPEs. E_{app} is 0.4 V vs pseudo RE, 12 mM glucose in phosphate buffer (100 mM, pH 7.5) was injected. The charges (Q) are the values after subtracting the background. Data are the average of n = 3 datasets, and error bars show standard deviation.

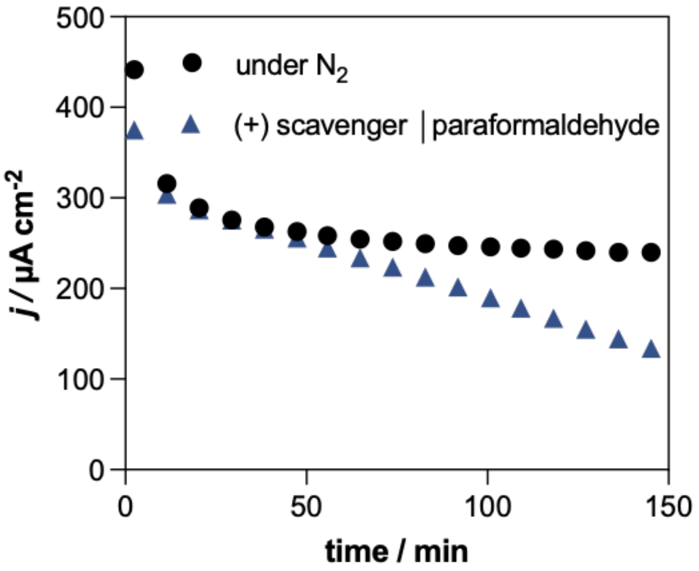

Fig. S20. Catalytic current density for lactate oxidation over time under N_2 and scavenger in phosphate buffer (pH 7.5, 100 mM, 1 mL) with 10 mM lactate. The oxygen scavenger contains paraformaldehyde (5 mg mL^{-1}), AOx (10 U mL^{-1}), and CAT (2000 U mL$^{-1)}$. Film composition: 50 wt% lactate oxidase 40 wt% Os-complex modified polymer, 10 wt% PEGDE with 500 µg cm^{-2} loading mass. The catalytic current density was measured from the CVs at a potential of 0.8 V *vs* SHE. GCEs were used as working electrodes for all experiments.

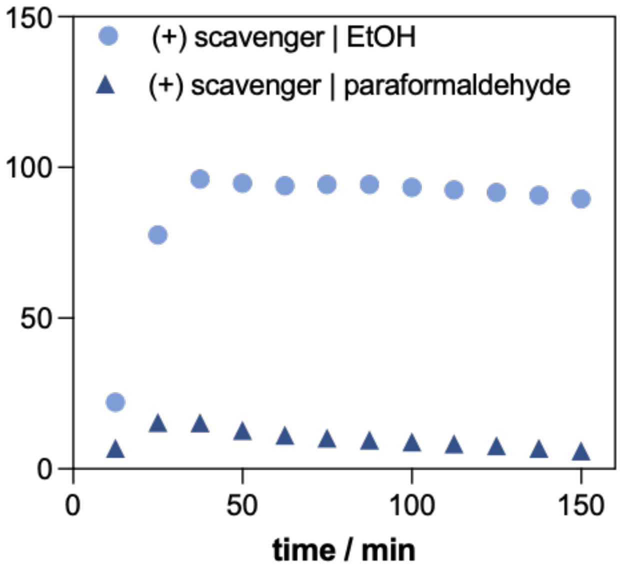

Fig. S21. Catalytic current density for creatinine oxidation over time in phosphate buffer (pH 7.5, 100 mM, 1 mL) with 25 mM creatinine, creatininase (60 U mL^{-1}) and creatinase (18 U mL^{-1}). The oxygen scavenger contains paraformaldehyde (5 mg mL^{-1}) or EtOH (50 mM), AOx (10 U mL^{-1}), and CAT (2000 U mL$^{-1)}$. Film composition: 46 wt% sarcosine oxidase, 46 wt% Os-complex modified polymer, 10 wt% PEGDE with 500 µg cm^{-2} loading mass. The catalytic current was measured from the CVs at a potential of 0.8 V *vs* SHE. GCEs were used as working electrodes for all experiments.

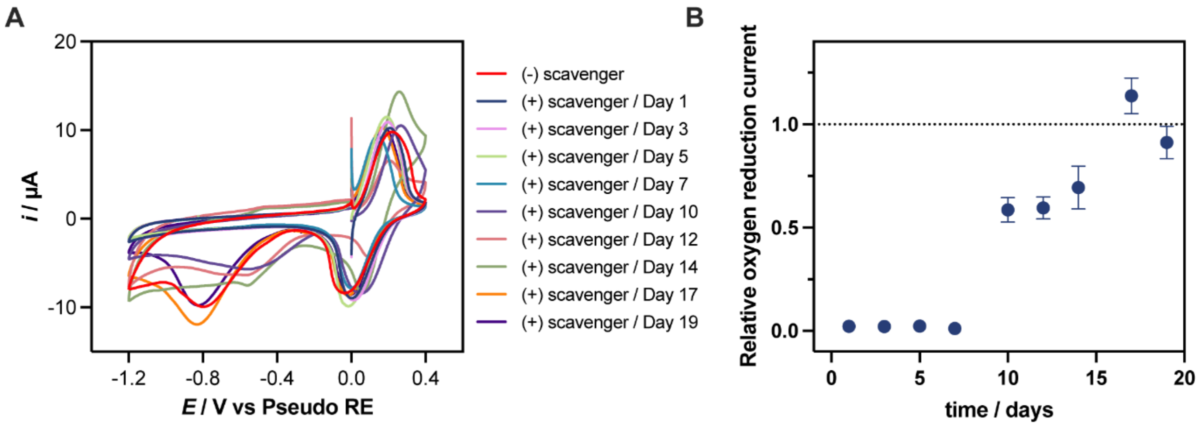

Fig. S22. Storage stability of AOx-based O_2 scavenger using paraformaldehyde as reducing agent. (A) CVs (10 mV s^{-1}) of SPEs modified with the Os-complex modified polymer in the presence of AOx / CAT / paraformaldehyde. CVs were taken after storage duration increasing from 1 to 19 days. **(B)** Normalized current *vs* the storage duration. The currents were measured at -0.8 V *vs* Pseudo RE. The current measured on Day 1 in the absence of the O_2 scavenger was used for normalization. All experiments were performed under air in phosphate buffer (pH 7.5, 100 mM), with the scavenger containing paraformaldehyde (50 mg mL^{-1}), AOx (10 U mL^{-1}), and CAT (2000 U $mL^{-1)}$. The SPEs were modified by drop-casting with Os-complex modified polymer (50 wt%), GOx (40 wt%) and PEGDGE (10 wt%) at a total loading of 50 µg cm^{-2}. SPEs were stored at 4 °C while exposed to air.

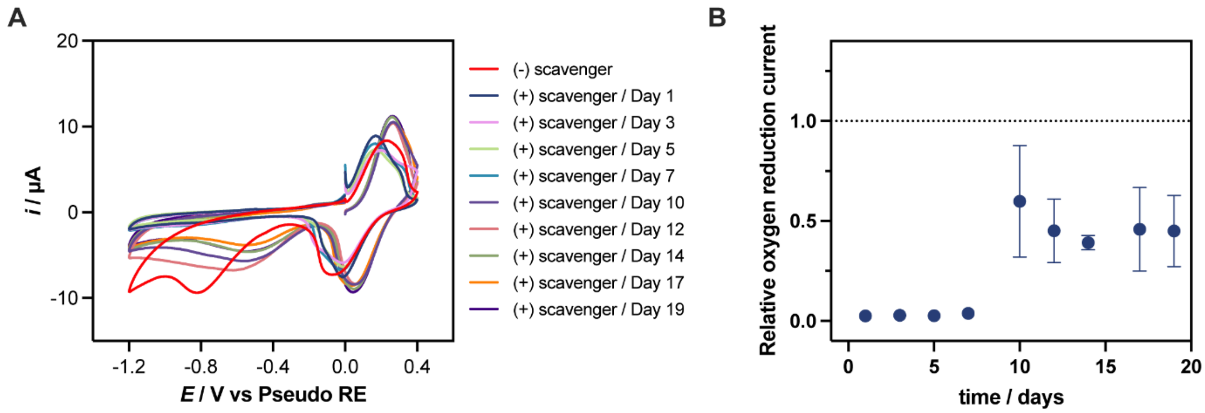

Fig. S23. Storage stability of AOx-based O_2 scavenger using ethanol as reducing agent. (A) CVs (10 mV s^{-1}) of SPEs modified with the Os-complex modified polymer in the presence of AOx / CAT / ethanol. CVs were taken after storage duration increasing from 1 to 19 days. **(B)** Normalized current *vs* the storage duration. The currents were measured at -0.8 V *vs* Pseudo RE. The current measured on Day 1 in the absence of the O_2 scavenger was used for normalization. All experiments were performed under air in phosphate buffer (pH 7.5, 100 mM), with the scavenger containing ethanol (250 mM), AOx (10 U mL^{-1}), and CAT (2000 U $mL^{-1)}$. The SPEs were modified by drop-casting with Os-complex modified polymer (50 wt%), GOx (40 wt%) and PEGDGE (10 wt%) at a total loading of 50 μg cm^{-2}. SPEs were stored at 4 °C while exposed to air.

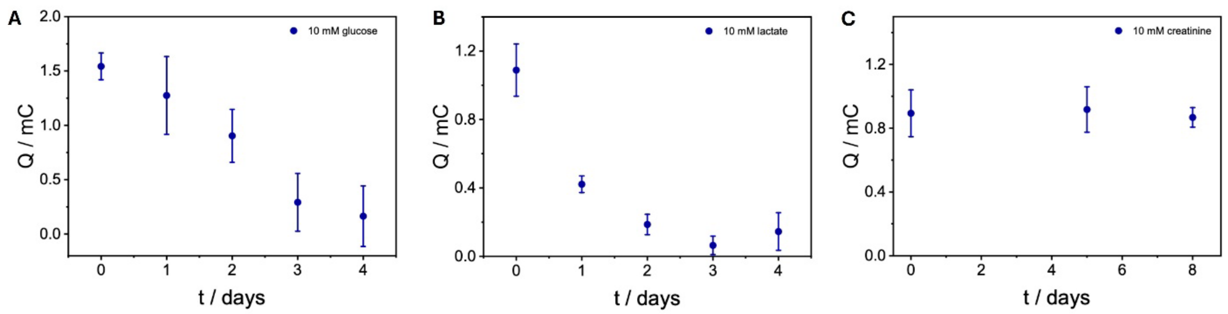

Fig. S24. Storage stability of the biosensors with integrated AOx-based O_2 scavenger. (A) glucose biosensors **(B)** lactate biosensors and **(C)** creatinine biosensors. For glucose biosensors and lactate biosensors, the scavenger containing AOx (40 U mL^{-1}), CAT (4000 U mL^{-1}) and paraformaldehyde (saturating concentrations ~50 mg mL^{-1}), was integrated within the capillary channel of the SPE. For the creatinine biosensor, ethanol (250 mM) was added to the electrolyte. For the glucose biosensor, the screen-printed electrodes were modified with Os-complex-modified polymer (50 wt%), GOx (40 wt%), and PEGDGE (10 wt%) at a total loading of 200 µg cm^{-2}. For the lactate biosensor, the screen-printed electrodes were modified with Os-complex-modified polymer (40 wt%), lactate oxidase (50 wt%), and PEGDGE (10 wt%) at a total loading of 50 µg cm^{-2}. For the creatinine biosensor, the screen-printed electrodes were modified with Os-complex-modified polymer (46 wt%), SOx (46 wt%), and PEGDGE (8 wt%) at a total loading of 50 µg cm^{-2}. Creatininase (2 mg mL^{-1}) and creatinase (4 mg mL^{-1}) were integrated within the capillary channel of the SPE. Data are the average of n = 3 repetitions and error bars show standard deviation.

	Pichia pastoris	*Candida boidinni*	*Hansenula polymorpha*
methanol	160.7 ± 8	39.86	24.1
ethanol	121.3 ± 12	40.54	22.1
propanol	63.0 ± 21	30.4	10.7
butanol	47.3 ± 12	29.7	12.0
pentanol	20.5 ± 7	10.8	0.7
formaldehyde	13.5 ± 0.1		

Data for AOx from *Pichia pastors* are the average of n = 3 datasets and error bars show standard deviation.

Table S1. k_{cat} (s^{-1}) of AOx from *Pichia pastors, Candida boidinni and Hansenula polymorpha*

Parameter	Quantity	Units
Distance to working electrode[a]	4	mm
Total length of capillary[a]	8	mm
Formaldehyde diffusion coefficient (D_F) (*65)*	$1.24 \cdot 10^{-5}$	$cm^2\ s^{-1}$
Oxygen diffusion coefficient (D_o) (*66)*	$2.4 \cdot 10^{-5}$	$cm^2\ s^{-1}$
Total formaldehyde concentration (C_F)[a]	10	mM
Total oxygen concentration (C_o)[a]	0.25	mM
Total enzyme concentration (C_E)[a]	20	µM
Catalytic kinetic constant (k_{cat})[b]	13.5	s^{-1}
Michaelis-Menten constant for oxygen (K_O) (*67*)	0.4	mM
Michaelis-Menten constant for formaldehyde (K_F)[b]	3.06	mM

a. The value was determined or estimated experimentally.

b. Values are in Table S1.

Table S2. Parameters for oxygen scavenging simulation with formaldehyde as the substrate

Parameter	Quantity	Units
Distance to working electrode[a]	4	mm
Total length of capillary[a]	8	mm
Ethanol diffusion coefficient (D_F) (*68*)	$1.2 \cdot 10^{-5}$	$cm^2\ s^{-1}$
Oxygen diffusion coefficient (D_o) (*66*)	$2.4 \cdot 10^{-5}$	$cm^2\ s^{-1}$
Total ethanol concentration (C_F)[a]	50	mM
Total oxygen concentration (C_o)[a]	0.25	mM
Total enzyme concentration (C_E)[a]	20	μM
Catalytic kinetic constant (k_{cat})[b]	121	s^{-1}
Michaelis-Menten constant for oxygen (K_O) (*67*)	0.4	mM
Michaelis-Menten constant for ethanol (K_{EtOH})[b]	6.4	mM

a. The value was determined or estimated experimentally.
b. Values are in Table S1.

Table S3. Parameters for oxygen scavenging simulation with ethanol as the substrate

	Chronocoulometric analysis			Amperometric analysis		
	Q / mC			i / µA		
T / °C	Mean	Standard deviation (±SD)	Relative SD / %	Mean	Standard deviation (±SD)	Relative SD / %
5	1.14	0.08	7.11	22.65	7.00	30.91
15	1.52	0.13	8.56	27.82	5.20	18.69
22	1.47	0.05	3.56	31.39	3.23	10.29
33	1.68	0.12	7.26	28.28	4.31	15.24
45	1.61	0.18	10.88	27.80	6.33	22.78
Relative SD average			7.47			19.58

Data are the average of n = 3 datasets and error bars show standard deviation.

Table S4. Chronocoulometric analysis vs amperometric analysis (at 3 s) of SPEs at different temperatures (5, 15, 22, 33, 45 °C) under ambient air.

REFERENCES AND NOTES

1. S. Li, H. Zhang, M. Zhu, Z. Kuang, X. Li, F. Xu, S. Miao, Z. Zhang, X. Lou, H. Li, F. Xia, Electrochemical biosensors for whole blood analysis: Recent progress, challenges, and future perspectives. *Chem. Rev.* **123**, 7953–8039 (2023).

2. P. Bollella, Enzyme-based amperometric biosensors: 60 years later … Quo Vadis? *Anal. Chim. Acta* **1234**, 340517 (2022).

3. R. Cai, C. Ngwadom, R. Saxena, J. Soman, C. Bruggeman, D. P. Hickey, R. Verduzco, C. M. Ajo-Franklin, Creation of a point-of-care therapeutics sensor using protein engineering, electrochemical sensing and electronic integration. *Nat. Commun.* **15**, 1689 (2024).

4. H. Teymourian, A. Barfidokht, J. Wang, Electrochemical glucose sensors in diabetes management: An updated review (2010–2020). *Chem. Soc. Rev.* **49**, 7671–7709 (2020).

5. J. Bai, D. Liu, X. Tian, Y. Wang, B. Cui, Y. Yang, S. Dai, W. Lin, J. Zhu, J. Wang, A. Xu, Z. Gu, S. Zhang, Coin-sized, fully integrated, and minimally invasive continuous glucose monitoring system based on organic electrochemical transistors. *Sci. Adv.* **10**, eadl1856 (2024).

6. J. Li, J. Liu, Z. Wu, X. Shang, Y. Li, W. Huo, X. Huang, Fully printed and self-compensated bioresorbable electrochemical devices based on galvanic coupling for continuous glucose monitoring. *Sci. Adv.* **9**, eadi3839 (2023).

7. Z. Pu, X. Zhang, H. Yu, J. Tu, H. Chen, Y. Liu, X. Su, R. Wang, L. Zhang, D. Li, A thermal activated and differential self-calibrated flexible epidermal biomicrofluidic device for wearable accurate blood glucose monitoring. *Sci. Adv.* **7**, eabd0199 (2021).

8. S. A. Pullano, M. Greco, M. G. Bianco, D. Foti, A. Brunetti, A. S. Fiorillo, Glucose biosensors in clinical practice: Principles, limits and perspectives of currently used devices. *Theranostics* **12**, 493–511 (2022).

9. A. Heller, B. Feldman, Electrochemical glucose sensors and their applications in diabetes management. *Chem. Rev.* **108**, 2482–2505 (2008).

10. R. N. Pittman, "Oxygen transport" in *Regulation of Tissue Oxygenation* (Morgan & Claypool Life Sciences, 2011).

11. N. Plumeré, J. Henig, W. H. Campbell, Enzyme-catalyzed O_2 removal system for electrochemical analysis under ambient air: Application in an amperometric nitrate biosensor. *Anal. Chem.* **84**, 2141–2146 (2012).

12. N. Plumeré, Interferences from oxygen reduction reactions in bioelectroanalytical measurements: The case study of nitrate and nitrite biosensors. *Anal. Bioanal. Chem.* **405**, 3731–3738 (2013).

13. T. Monteiro, P. R. Rodrigues, A. L. Gonçalves, J. J. Moura, E. Jubete, L. Añorga, B. Piknova, A. N. Schechter, C. M. Silveira, M. G. Almeida, Construction of effective disposable biosensors for point of care testing of nitrite. *Talanta* **142**, 246–251 (2015).

14. T. Monteiro, M. Moreira, S. B. R. Gaspar, M. G. Almeida, Bilirubin oxidase as a single enzymatic oxygen scavenger for the development of reductase-based biosensors in the open air and its application on a nitrite biosensor. *Biosens. Bioelectron.* **217**, 114720 (2022).

15. A. F. T. Waffo, B. Mitrova, K. Tiedemann, C. Iobbi-Nivol, S. Leimkühler, U. Wollenberger, Electrochemical trimethylamine N-oxide biosensor with enzyme-based oxygen-scavenging membrane for long-term operation under ambient air. *Biosensors* **11**, 98 (2021).

16. H.-c. Cheng, M. Abo, A. Okubo, Development of dimethyl sulfoxide biosensor using a mediator immobilized enzyme electrode. *Analyst* **128**, 724–727 (2003).

17. F. Ricci, F. Caprio, A. Poscia, F. Valgimigli, D. Messeri, E. Lepori, G. Dall'Oglio, G. Palleschi, D. Moscone, Toward continuous glucose monitoring with planar modified biosensors and microdialysis. Study of temperature, oxygen dependence and in vivo experiment. *Biosens. Bioelectron.* **22**, 2032–2039 (2007).

18. A. Prévoteau, N. Mano, Oxygen reduction on redox mediators may affect glucose biosensors based on "wired" enzymes. *Electrochim. Acta* **68**, 128–133 (2012).

19. H. M. Bambhania, D. Chakraborty, H. Wen, S. C. Barton, Impact of oxygen on glucose oxidation kinetics in a redox polymer mediated glucose oxidase electrode. *J. Electrochem. Soc.* **164**, H232–H240 (2017).

20. N. Mano, F. Mao, A. Heller, On the parameters affecting the characteristics of the "wired" glucose oxidase anode. *J. Electroanal. Chem.* **574**, 347–357 (2005).

21. G. Kopiec, K. Starzec, J. Kochana, T. P. Kinnunen-Skidmore, W. Schuhmann, W. H. Campbell, A. Ruff, N. Plumeré, Bioelectrocatalytic and electrochemical cascade for phosphate sensing with up to 6 electrons per analyte molecule. *Biosens. Bioelectron.* **117**, 501–507 (2018).

22. M. E. Rollie, G. Patonay, I. M. Warner, Deoxygenation of solutions and its analytical applications. *Ind. Eng. Chem. Res.* **26**, 1–6 (1987).

23. I. B. Butler, M. A. Schoonen, D. T. Rickard, Removal of dissolved oxygen from water: A comparison of four common techniques. *Talanta* **41**, 211–215 (1994).

24. T. M. Florence, Y. J. Farrar, Removal of oxygen from polarographic solutions with ascorbic acid. *J. Electroanal. Chem. Interfacial Electrochem.* **41**, 127–133 (1973).

25. Y. Gu, C.-C. Chen, Eliminating the interference of oxygen for sensing hydrogen peroxide with the polyaniline modified electrode. *Sensors* **8**, 8237–8247 (2008).

26. Y. Israel, A. Vromen, B. Paschkes, Polarographic study of inhibitors and catalysts for the reaction of dissolved oxygen with sulphite ion. *Talanta* **14**, 925–931 (1967).

27. A. M. Pisoschi, A. Pop, A. I. Serban, C. Fafaneata, Electrochemical methods for ascorbic acid determination. *Electrochim. Acta* **121**, 443–460 (2014).

28. R. E. Benesch, R. Benesch, Enzymatic removal of oxygen for polarography and related methods. *Science* **118**, 447–448 (1953).

29. S. W. Englander, D. B. Calhoun, J. J. Englander, Biochemistry without oxygen. *Anal. Biochem.* **161**, 300–306 (1987).

30. M. Swoboda, J. Henig, H.-M. Cheng, D. Brugger, D. Haltrich, N. Plumeré, M. Schlierf, Enzymatic oxygen scavenging for photostability without pH drop in single-molecule experiments. *ACS Nano* **6**, 6364–6369 (2012).

31. H. Yonehara, S.-i. Fujii, K. Sato, M. Abo, E. Yoshimura, Construction of a dimethyl sulfoxide sensor based on dimethyl sulfoxide reductase immobilized on a Au film electrode. *Anal. Sci.* **23**, 55–58 (2007).

32. G. Wohlfahrt, S. Trivić, J. Zeremski, D. Peričin, V. Leskovac, The chemical mechanism of action of glucose oxidase from Aspergillus niger. *Mol. Cell. Biochem.* **260**, 69–83 (2004).

33. J. Jordan, M. K. Ciolkosz, Enzymatic mechanisms and electron transfer mediation in chronoamperometric biosensors. *J. Solution Chem.* **20**, 995–1000 (1991).

34. D. P. Hickey, R. C. Reid, R. D. Milton, S. D. Minteer, A self-powered amperometric lactate biosensor based on lactate oxidase immobilized in dimethylferrocene-modified LPEI. *Biosens. Bioelectron.* **77**, 26–31 (2016).

35. R. N. Patel, C. T. Hou, A. I. Laskin, P. Derelanko, Microbial oxidation of methanol: Properties of crystallized alcohol oxidase from a yeast, Pichia sp. *Arch. Biochem. Biophys.* **210**, 481–488 (1981).

36. K. G. Helander, Formaldehyde prepared from paraformaldehyde is stable. *Biotech. Histochem.* **75**, 19–22 (2000).

37. C. F. Breitkreuz, J. Burger, H. Hasse, Solid–liquid equilibria and kinetics of the solid formation in binary and ternary mixtures containing (formaldehyde + water + methanol). *Ind. Eng. Chem. Res.* **61**, 1871–1884 (2022).

38. S. Ferri, K. Kojima, K. Sode, Review of glucose oxidases and glucose dehydrogenases: A bird's eye view of glucose sensing enzymes. *J. Diabetes Sci. Technol.* **5**, 1068–1076 (2011).

39. J. Wang, Electrochemical glucose biosensors. *Chem. Rev.* **108**, 814–825 (2008).

40. A. Prévoteau, N. Mano, How the reduction of O_2 on enzymes and/or redox mediators affects the calibration curve of “wired” glucose oxidase and glucose dehydrogenase biosensors. *Electrochim. Acta* **112**, 318–326 (2013).

41. C. Schmid, A. Baumstark, S. Pleus, C. Haug, M. Tesar, G. Freckmann, Impact of partial pressure of oxygen in blood samples on the performance of systems for self-monitoring of blood glucose. *Diabetes Technol. Ther.* **16**, 156–165 (2013).

42. L. Lei, C. Xu, X. Dong, B. Ma, Y. Chen, Q. Hao, C. Zhao, H. Liu, Continuous glucose monitoring in hypoxic environments based on water splitting-assisted electrocatalysis. *Chem* **11**, 149 (2023).

43. R. J. Forster, J. G. Vos, Synthesis, characterization, and properties of a series of osmium-and ruthenium-containing metallopolymers. *Macromolecules* **23**, 4372–4377 (1990).

44. J. M. González Lara, F. P. Cardona, A. R. Vallmajor, M. C. Cadevall, Oxidation of thiosulfate with oxygen using copper (II) as a catalyst. *Metals* **9**, 387 (2019).

45. T. J. Ohara, R. Rajagopalan, A. Heller, Glucose electrodes based on cross-linked bis(2,2'-bipyridine)chloroosmium(+/2+) complexed poly(1-vinylimidazole) films. *Anal. Chem.* **65**, 3512–3517 (1993).

46. J. Bao, K. Furumoto, M. Yoshimoto, K. Fukunaga, K. Nakao, Competitive inhibition by hydrogen peroxide produced in glucose oxidation catalyzed by glucose oxidase. *Biochem. Eng. J.* **13**, 69–72 (2003).

47. K. Kleppe, The effect of hydrogen peroxide on glucose oxidase from Aspergillus niger*. *Biochemistry* **5**, 139–143 (1966).

48. T. Von Woedtke, U. Fischer, P. Abel, Glucose oxidase electrodes: Effect of hydrogen peroxide on enzyme activity? *Biosens. Bioelectron.* **9**, 65–71 (1994).

49. T. K. Mathew, M. Zubair, P. Tadi, “Blood glucose monitoring” in *StatPearls* (StatPearls Publishing, 2024).

50. M. I. Friedman, A. L. Emmerich, K. M. Gil, Effects of insulin on food intake and plasma glucose level in fat-fed diabetic rats. *Physiol. Behav.* **24**, 319–325 (1980).

51. K. R. Temsamani, K. L. Cheng, Studies of chloride adsorption on the Ag/AgCl electrode. *Sens. Actuators B Chem.* **76**, 551–555 (2001).

52. R. K. Rakesh Kumar, M. O. Shaikh, C. H. Chuang, A review of recent advances in non-enzymatic electrochemical creatinine biosensing. *Anal. Chim. Acta* **1183**, 338748 (2021).

53. F. Alam, S. RoyChoudhury, A. H. Jalal, Y. Umasankar, S. Forouzanfar, N. Akter, S. Bhansali, N. Pala, Lactate biosensing: The emerging point-of-care and personal health monitoring. *Biosens. Bioelectron.* **117**, 818–829 (2018).

54. K. Hiraka, K. Kojima, C.-E. Lin, W. Tsugawa, R. Asano, J. T. La Belle, K. Sode, Minimizing the effects of oxygen interference on l-lactate sensors by a single amino acid mutation in Aerococcus viridans l-lactate oxidase. *Biosens. Bioelectron.* **103**, 163–170 (2018).

55. Q. Zhou, C.-P. Chng, Y. Zhao, Y. Wang, H. Xu, Y. Huo, C. Huang, Ethanol-induced gelation enables direct three-dimensional printing of sodium alginate hydrogel. *Mater. Des.* **239**, 112746 (2024).

56. C.-P. Chang, W.-C. Fann, S.-R. Wu, C.-N. Lin, C.-T. Hsiao, Lactate on emergency department arrival as a predictor of in-hospital mortality in necrotizing fasciitis: A retrospective study. *J. Orthop. Surg. Res.* **14**, 73 (2019).

57. C. S. Pundir, P. Kumar, R. Jaiwal, Biosensing methods for determination of creatinine: A review. *Biosens. Bioelectron.* **126**, 707–724 (2019).

58. E. Tremey, E. Suraniti, O. Courjean, S. Gounel, C. Stines-Chaumeil, F. Louerat, N. Mano, Switching an O_2 sensitive glucose oxidase bioelectrode into an almost insensitive one by cofactor redesign. *Chem Commun.* **50**, 5912–5914 (2014).

59. Y. Horaguchi, S. Saito, K. Kojima, W. Tsugawa, S. Ferri, K. Sode, Engineering glucose oxidase to minimize the influence of oxygen on sensor response. *Electrochim. Acta* **126**, 158–161 (2014).

60. M. Grattieri, M. Tucci, M. Bestetti, S. Trasatti, P. Cristiani, Facilitated electron hopping in nanolayer oxygen-insensitive glucose biosensor for application in a complex matrix. *ChemElectroChem* **3**, 1884–1889 (2016).

61. F. Lopez, S. Zerria, A. Ruff, W. Schuhmann, An O_2 tolerant polymer/glucose oxidase based bioanode as basis for a self-powered glucose sensor. *Electroanalysis* **30**, 1311–1318 (2018).

62. I. Lee, D. Probst, D. Klonoff, K. Sode, Continuous glucose monitoring systems - Current status and future perspectives of the flagship technologies in biosensor research. *Biosens. Bioelectron.* **181**, 113054 (2021).

63. K. Rungsrisuriyachai, G. Gadda, A pH switch affects the steady-state kinetic mechanism of pyranose 2-oxidase from Trametes ochracea. *Arch. Biochem. Biophys.* **483**, 10–15 (2009).

64. D. Buesen, H. Li, N. Plumeré, The electron as a probe to measure the thickness distributions of electroactive films. *Chem. Sci.* **11**, 937–946 (2020).

65. S. Tolosa, J. A. Sansón, Molecular dynamics study of infinitely dilute aqueous solutions of small biological molecules. Calculation of the static and dynamic properties of formaldehyde. *Chem. Phys.* **213**, 203–210 (1996).

66. J. E. Vivian, C. J. King, The mechanism of liquid-phase resistance to gas absorption in a packed column. *AIChE J.* **10**, 221–227 (1964).

67. R. Couderc, J. Baratti, Oxidation of methanol by the yeast, Pichia pastoris. Purification and properties of the alcohol oxidase. *Agric. Biol. Chem.* **44**, 2279–2289 (1980).

68. L. Hao, D. G. Leaist, Binary mutual diffusion coefficients of aqueous alcohols. Methanol to 1-heptanol. *J. Chem. Eng. Data* **41**, 210–213 (1996).
